# Pharmacometrics-Enhanced Bayesian Borrowing for Pediatric Extrapolation – A Case Study of the DINAMO Trial

**DOI:** 10.1007/s43441-024-00707-5

**Published:** 2024-10-07

**Authors:** Martin Oliver Sailer, Dietmar Neubacher, Curtis Johnston, James Rogers, Matthew Wiens, Alejandro Pérez-Pitarch, Igor Tartakovsky, Jan Marquard, Lori M. Laffel

**Affiliations:** 1 Global Biostatistics & Data Sciences, Boehringer Ingelheim Pharma GmbH & Co. KG, Birkendorfer Strasse 65, 88397 Biberach, Germany; 2 Metrum Research Group, Tariffville, CT 06081 USA; 3 Boehringer Ingelheim Pharma GmbH & Co. KG, Ingelheim, Germany; 4 Boehringer Ingelheim Pharmaceuticals Inc, Ridgefield, CT 06877 USA; 5 Joslin Diabetes Center, Harvard Medical School, Boston, MA USA; 6 Present Address: Regeneron Pharmaceuticals Inc, Tarrytown, NY USA

**Keywords:** Bayesian dynamic borrowing, Pediatric extrapolation, Empagliflozin, Linagliptin, Modeling & simulation

## Abstract

Bayesian borrowing analyses have an important role in the design and analysis of pediatric trials. This paper describes use of a prespecified Pharmacometrics Enhanced Bayesian Borrowing (PEBB) analysis that was conducted to overcome an expectation for reduced statistical power in the pediatric DINAMO trial due to a greater than expected variability in the primary endpoint. The DINAMO trial assessed the efficacy and safety of an empagliflozin dosing regimen versus placebo and linagliptin versus placebo on glycemic control (change in HbA1c over 26 weeks) in young people with type 2 diabetes (T2D). Previously fitted pharmacokinetic and exposure-response models for empagliflozin and linagliptin based on available historical data in adult and pediatric patients with T2D were used to simulate participant data and derive the informative component of a Bayesian robust mixture prior distribution. External experts and representatives from the U.S. Food and Drug Administration provided recommendations to determine the effective sample size of the prior and the weight of the informative prior component. Separate exposure response-based Bayesian borrowing analyses for empagliflozin and linagliptin showed posterior mean and 95% credible intervals that were consistent with the trial results. Sensitivity analyses with a full range of alternative weights were also performed. The use of PEBB in this analysis combined advantages of mechanistic modeling of pharmacometric differences between adults and young people with T2D, with advantages of partial extrapolation through Bayesian dynamic borrowing. Our findings suggest that the described PEBB approach is a promising option to optimize the power for future pediatric trials.

**Supplementary Information:**

The online version contains supplementary material available at 10.1007/s43441-024-00707-5.

## Introduction

The sodium-glucose transport protein 2 (SGLT2) inhibitor empagliflozin and the dipeptidyl peptidase 4 (DPP-4) inhibitor linagliptin are well-established treatments for adults with type 2 diabetes (T2D) [1]. In children and adolescents, treatment options are more limited [2]. Until recently, oral metformin and insulin were the only approved treatments. To overcome this limitation, the DIabetes study of liNAgliptin and eMpagliflozin in children and adOlescents (DINAMO) trial was conducted to evaluate the efficacy and safety of empagliflozin and linagliptin in a pediatric sample.

DINAMO was a 26-week, double-blind, randomized, placebo-controlled trial with a safety extension period of an additional 26 weeks in 157 pediatric T2D participants aged 10 to 17 years [3]. Participants were randomized to placebo, linagliptin 5 mg, or empagliflozin 10 mg. Those randomized to empagliflozin 10 mg who did not achieve an HbA1c < 7% at week 12 were re-randomized at week 14 to either continue empagliflozin 10 mg or begin empagliflozin 25 mg. The primary efficacy endpoint was change from baseline in hemoglobin A1c (HbA1c) at 26 weeks, tested simultaneously for the pooled empagliflozin dosing group (including all who received empagliflozin at any dose) versus placebo and for linagliptin versus placebo.

Using a balance of clinical, regulatory, feasibility, and statistical considerations, we aimed to enroll 50 participants per group [3]. Based on previous trials of empagliflozin or linagliptin in adults with T2D receiving background medication of metformin or insulin, the mean differences in the HbA1c change from baseline to week 26 in the treatment groups versus placebo group were estimated as − 0.55% with a standard deviation (SD) of 0.9% [3]. With 50 participants per treatment group, a treatment difference of − 0.55% could be detected with 85% power at a two-sided α level of 5%.

After trial recruitment was completed, a blinded assessment of the variability of the primary endpoint in 141 participants from DINAMO revealed a SD for the primary endpoint of 1.65%, higher than the anticipated covariate-adjusted SD of 0.9% used in sample size calculations. Consequently, there would be reduced statistical power for the primary analysis unless a greater treatment effect than anticipated occurred. To address potentially reduced power, we performed a prespecified Bayesian borrowing analysis of DINAMO to generate supportive evidence for effectiveness of empagliflozin and linagliptin.

Bayesian statistics enables data from previous trials to directly contribute to analysis of data from a new clinical trial [4]. The Bayesian approach uses Bayes’ theorem to formally combine prior information with current information on a measure of interest such as treatment effect [5]. This prior information is represented as a probability distribution (prior distribution), which is then updated with data from a new study to obtain a “posterior distribution” for the measure of interest (treatment effect) that combines the two sources of evidence. The Bayesian posterior thereby borrows prior information to augment the evidence from a new study.

Standard Bayesian borrowing analysis assumes marginal (i.e., not covariate-adjusted) exchangeability of historical prior information and new study data. In the present situation, marginal exchangeability of adult HbA1c data with pediatric HbA1c data from DINAMO is not plausible due to expected differences in covariate constellations (e.g. distribution of race, body weight, renal function, or diabetes duration), but covariate-adjusted exchangeability [6] (i.e. similarity after accounting for known physiological and pharmacological differences) is plausible. As implemented in our analysis, Bayesian dynamic borrowing then accommodates the possibility that exchangeability is not achieved even after covariate adjustment, by providing a statistically rigorous mechanism to down-weight prior information that is not consistent with new study data [4]. Bayesian dynamic borrowing is used increasingly in clinical drug development, in line with current regulatory guidance [7–13].

This paper provides an overview of a Bayesian borrowing approach used to provide additional supportive evidence for effectiveness of empagliflozin and linagliptin, increasing confidence in the DINAMO results. We highlight use of a pharmacometrics-based model approach, known as Pharmacometrics Enhanced Bayesian Borrowing (PEBB) [14], to leverage data from adult trials. The informative component of Bayesian prior distributions is derived from fitted population pharmacokinetic (PopPK) and exposure-response models for empagliflozin and linagliptin based on available historical data in adult and pediatric patients with T2D.

## Materials and Methods

### Overview

**Fig. 1:**
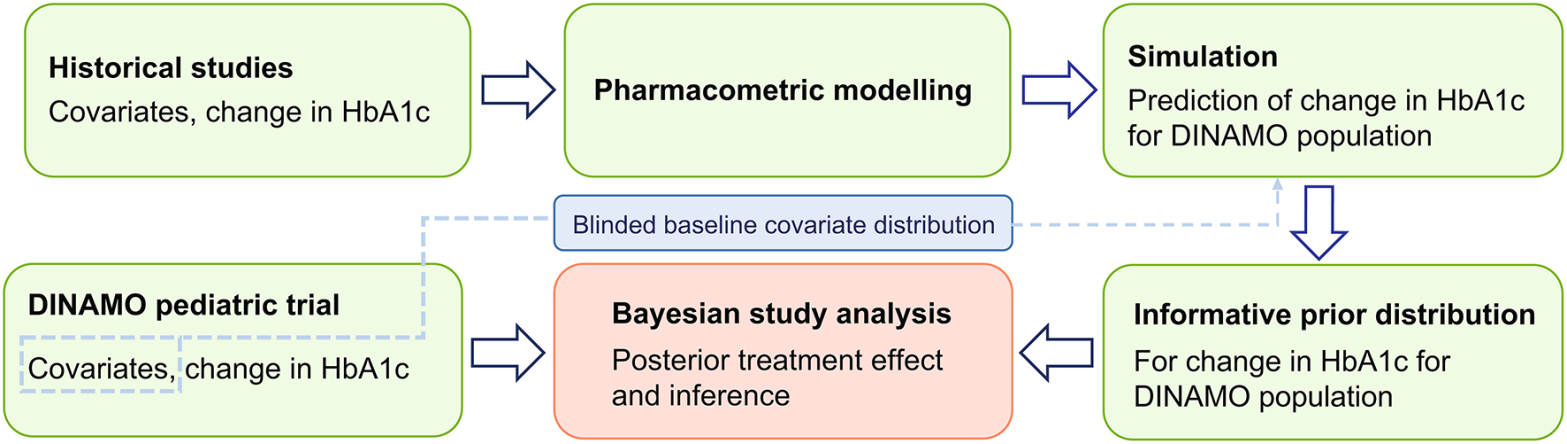
Schematic workflow of Bayesian borrowing analysis

The PEBB framework in this analysis followed the steps described in Fig. 1. First, pharmacometric modeling of the exposure-response relationship of empagliflozin and linagliptin was performed based on available historical data in adults and pediatric patients with T2D. Second, these models simulated individual and placebo-corrected mean changes in HbA1c at 26 weeks in a pediatric population with baseline characteristics matching the DINAMO population in relevant covariates. Third, the prior distribution was robustified with a weakly-informative mixture component to allow for the possibility of prior-data conflict. Prior weights for the informative and non-informative components were determined in advance based on feedback from clinical experts and the U.S. Food and Drug Administration (FDA) and were constrained to yield prior distributions with effective sample sizes (ESS) less than or equal to the observed sample sizes in DINAMO. Fourth, the posterior distribution was derived, combining the prior distribution with the DINAMO results. The prior was automatically updated based on data for the posterior distribution. Fifth, the posterior distribution was summarized, providing an estimate of the treatment effect that represents the totality of evidence from both DINAMO data and historical data. Finally, sensitivity analyses were performed using alternative weights for the informative component of the prior.

### Data Sources and Primary Analysis

Data used for the empagliflozin pharmacometric models included 14 phase II and III trials (Table S1). Data for the linagliptin pharmacometric models included 8 phase II and III trials (Table S2). Of these 22 trials, 20 were conducted in adults with 2 pediatric trials [15, 16], including 66 participants. In the DINAMO trial, a total of 157 participants received either empagliflozin (10 mg or 25 mg; *n* = 52), linagliptin 5 mg (*n* = 52), or placebo (*n* = 53) [3]. The primary DINAMO trial analysis was performed with an analysis of covariance (ANCOVA) adjusted for treatment, baseline HbA1c, and baseline age group. At week 26, treatment with linagliptin did not provide a significant improvement in HbA1c compared with placebo [placebo-adjusted treatment difference − 0.34% (95% confidence interval [CI] − 0.99 to 0.30; *p* = 0.29)]; however, treatment with empagliflozin was superior in reducing HbA1c from baseline versus placebo [placebo-adjusted treatment difference − 0.84% (95% CI − 1.50 to − 0.19, *p* = 0.012)].

### Software for Analyses

Software details for the pharmacometric models and Bayesian borrowing analyses appear in Supplementary Materials.

### Pharmacometric Models

All pharmacometric models were prespecified prior to unblinding of DINAMO data.

#### PK Sampling and Bioanalytical Assays

Details of the blood sampling and bioanalytical methods for measurement of empagliflozin and linagliptin plasma concentrations used in PopPK models appear in Supplementary Materials and Table S3.

#### Empagliflozin PopPK Model Development

A previously developed two-compartmental PopPK model in adults with T2D [17] was updated for use in the pediatric population. Three additional trials were included [15, 18, 19], with a fourth trial [20] providing external validation. Some structural simplifications were made regarding absorption and covariate inclusion. Observed absorption lag, evident in some participants, was modeled with a sequential zero-first-order absorption process, rather than the discontinuous absorption lag time (ALAG) parameter previously used. Given limited number of laboratory measures collected in the single pediatric PK trial [15], a limited number of covariates were analyzed (Table S4). Allometric scaling with body weight was added into the model to facilitate translation of the PopPK model between adult and pediatric groups to account for changes in clearance and volume due to potential differences in weight for pediatric patients [21]. Further information on model development appears in Supplementary Materials.

#### Empagliflozin PK-PD Model Development

A previous pharmacokinetic-pharmacodynamic (PK-PD) model in adults [17] was updated for the pediatric population. As fasting plasma glucose (FPG) was not densely sampled in the single included pediatric PK trial [15], the updated model removed FPG which was previously an intermediary between empagliflozin exposure (AUC) and HbA1c changes. Covariate relationships were pre-specified (Table S4) with the updated model incorporating effects of concomitant medications (insulin, metformin, and sulfonylurea) commonly used in pediatric patients with T2D.

An indirect response model described the effect of empagliflozin exposure on HbA1c. HbA1c modeling used a turnover model parameterized as baseline HbA1c, HbA1c synthesis rate constant (k_in_), an HbA1c first-order degradation rate constant (k_out_), with empagliflozin inhibiting the k_in_ parameter through a maximum inhibition (*I*_*max*_) relationship. A placebo effect was also incorporated, which affected the k_out_ parameter. Area under the concentration-time curve for a dosing interval at steady state (AUC_ss_) producing half-maximal inhibitory effect (AUC_50_) for the drug effect was fixed to 703 nmol·h/L, the value estimated in a previous PK-PD model that included FPG [17]. Covariates selected for evaluation included background medications used in DINAMO participants, renal function, and baseline HbA1c (Table S4). Further information on model development appears in Supplementary Materials.

#### Linagliptin PopPK Model Development

A previously developed two-compartmental PopPK model in adults with T2D [22–24] was updated to be more parsimonious and enable accommodation of PK data from pediatric participants. Due to the non-linear PK of linagliptin, there was difficulty in modeling all available linagliptin PK data (early and late phase studies). This was overcome by using a subset of data (the analysis-model dataset) from 5 well-controlled, early-phase trials [16, 25–28] to characterize the structural model and for parameter estimation.

A limited number of covariates were analyzed in the analysis-model dataset (Table S4), with the final model using a greater number of covariates to generate maximum *a posteriori* (MAP) Bayes parameter predictions for a broader dataset that included another 3 linagliptin trials [29–31] (known as the empirical Bayes estimates (EBE)-model dataset) to obtain exposures for HbA1c modeling. Further information on model development appears in Supplementary Materials.

#### Linagliptin PK-PD Model Development

As a starting point, the exposure-response relationship between HbA1c lowering and linagliptin exposure used a turnover model for HbA1c. AUC_ss_ provided the exposure response relationship; with sequential modeling of placebo and exposure responses implemented as necessary (See Supplementary Materials for further details).

Data from all trials was used in model development except in those where HbA1c was not collected [25]. Covariates selected for evaluation included background medications (metformin, insulin) in anticipation of the DINAMO sample (Table S4).

#### Model Selection and Evaluation

Standard model selection and discrimination criteria were used in the development of pharmacometric models (see Supplementary Materials for further details). Models providing best description of data without any unacceptable trends in goodness-of-fit (GOF) plots were used [32]. Final pharmacometric model parameter estimates were reported with an estimation measure of uncertainty including 95% credible intervals (CDIs) from the posterior parameter distributions of Bayes runs and 95% CIs derived from asymptotic standard error estimates or non-parametric bootstrap.

The final PopPK and PK-PD models were qualified by inspection of longitudinal visual predictive checks (VPCs) [33], prediction-corrected VPCs (pc-VPCs) [33], and by NPDE [34] plots (see Supplementary Materials for further details). The impact of covariates on PopPK and PK-PD models was assessed by examining forest plots of changes in apparent clearance after oral dosing (CL/F) and HbA1c change from baseline, respectively.

#### Model Simulations

We conducted 5,000 simulations using the developed PopPK and PK-PD models for empagliflozin and linagliptin in an iterative manner for a total of 5,000 iterations. In each iteration, 5,000 patients (empagliflozin and linagliptin) were resampled without stratification using blinded demographic and background medication data from DINAMO (158 randomized participants). The simulations mimicked the design specifications of DINAMO (treatment groups and re-randomization at week 14) and generated placebo-corrected mean changes from baseline for HbA1c (%) to serve as prior information for the Bayesian borrowing analyses. Further details about simulations appear in Supplementary Materials.

### Bayesian Borrowing Analysis Based on Exposure-Response Model

#### Prior Calculation

Bayesian inference compared the mean change in HbA1c from baseline to week 26 between empagliflozin and placebo, as well as between linagliptin and placebo. This is referred to as the placebo-corrected treatment effect of empagliflozin and linagliptin, respectively. Partial exchangeability of relevant historical, mostly adult clinical trial data informed the treatment comparison and increased the precision of estimates.

To minimize bias of partial exchangeability, the PK-PD models described above in adults generated a covariate-adjusted mean prediction of HbA1c responses in a pediatric population using the participant characteristics of DINAMO. This was considered acceptable based on the confidence in describing differences in exposure after allometric scaling by weight and previous comparable responses for short-term markers of efficacy (e.g. urinary glucose excretion [UGE] for empagliflozin [Boehringer Ingelheim, data on file], and DPP-4 inhibition for linagliptin [35]) in pediatric patients with T2D relative to adults.

Random samples of the predicted placebo-corrected treatment effect distributions informed the prior distributions for Bayesian analysis. To reduce the impact of potential discrepancies between historical and new HbA1c predictions and prevent prior-data conflict, the prior distribution was robustified by adding a weakly-informative mixture component to the calculated prior [36]. The robust component of the prior dynamically down weights the informative component of the prior (i.e., that derived from adult pharmacometric models) in cases of prior-data conflict. Separate analyses were performed for empagliflozin and linagliptin.

Since pharmacometric model predictions were based on predominantly adult participants with limited pediatric data, there was uncertainty about the information content in the pharmacometric model predictions. Expert input from members of the DINAMO steering committee helped to assess the ESS of the prior and weight of the informative part of the prior. The experts determined that an ESS of at most 100 participants per treatment group reflected a consensus degree of confidence in the covariate-adjusted pharmacometric model predictions. If the ESS implied from the pharmacometric simulation was greater than 100, then the variance of the informative prior would be down-weighted accordingly to correspond to an ESS of 100. This was in line with recommendations to derive priors that give the data a chance to influence the outcome of a trial in a pediatric setting where adult data may otherwise outweigh the pediatric data [37].

Based on feedback from the FDA, the maximum number of effective borrowed patients as determined by the calculated ESS of the prior distribution (as opposed to the ESS for the informative component) was adjusted to be no more than the sample size of each treatment group of DINAMO (~ 52 patients per treatment group). To fully utilize the informative part of the prior up to the limit defined by FDA, the (pre-specified) weight for the informative part of the prior was set to 0.65 (with a non-informative weight 0.35), resulting in an approximate overall ESS of the prior of 51 patients per treatment group (i.e., less than the total number of enrolled participants in each treatment group). Sensitivity analyses were conducted using a full range of alternative weights.

ESS was calculated with the expected local information ratio (ELIR) method [38]. Further details and key equations for this section appear in Supplementary Materials.

#### Posterior Calculation and Decision Rule

The posterior distributions were used to describe and evaluate the placebo corrected treatment effects for empagliflozin and linagliptin, separately, and to perform statistical inference. The posterior distribution of the treatment effect was calculated from the prior and the observed placebo-corrected treatment effect in DINAMO.

The pre-specified decision rule to determine efficacy of each treatment was based on comparison of the 97.5% quantile of the posterior treatment effect with 0. If the decision criterion was met (i.e., posterior distribution value for 97.5% quantile was less than 0), then there was evidence of superior efficacy of each treatment in the DINAMO sample (See Supplementary Materials for further details).

#### Operating Characteristics

The frequentist operating characteristics (i.e., power and type I error) for the Bayesian borrowing analysis were calculated for a set of scenarios with varying assumptions about the true placebo-corrected treatment effect in the DINAMO trial. They were also calculated for alternative choices of the informative prior part weight and ESS to assess robustness of the choice of weight and ESS [39]. As shown in Table S5, using prior means for empagliflozin ($\:{\mu}_{E}$) and linagliptin ($\:{\mu}_{L}$) equal to − 0.55, an informative part ESS of 100 per treatment group and informative prior weights for empagliflozin ($\:{w}_{E}$) and linagliptin ($\:{w}_{L}$) equal to 0.65, a good trade-off is obtained between power gains through borrowing and the need to limit the type I error inflation. With these operating characteristics, the probability of meeting the decision criterion with evidence level of 97.5% is 79.3% for empagliflozin and 76.9% for linagliptin (scenario 10 in Table S5). This scenario corresponds to the placebo-corrected treatment effect assumed in the sample size calculation of DINAMO. This represents a considerable improvement over the 40.1% (empagliflozin) and 37.7% (linagliptin) probability at the 97.5% evidence level where a weakly informative prior ($\:{w}_{E}$ = $\:{w}_{L}$ = 0) is used (scenario 18 in TableS5). The use of the informative prior increases also the false positive decision probability. For example, there is a 19.0% (empagliflozin) and 18.5% (linagliptin) probability of falsely meeting the decision criterion when there is no treatment benefit (scenario 12 in Table S5). This increase beyond the significance level chosen in the DINAMO protocol was considered acceptable (See Supplementary Materials, Tables  S5/S6 and Figures S2/ S3 for further details).

#### Sensitivity Analyses

We performed a tipping point sensitivity analysis with alternative prior weights (range 0 to 1) for the informative component [40] (see Supplementary Materials for further details). In sensitivity analyses for the posterior, inference was also performed with lower decision rule thresholds (95% and 90% quantiles).

## Results

### Pharmacometric Models

#### Empagliflozin PopPK Model

The empagliflozin PopPK model dataset included 5384 participants from 14 trials (Table S1) with a total of 19,473 observed PK concentrations, ~ 3.5% of which were below the LLOQ. Doses in these trials ranged from 1 − 100 mg, with 10 and 25 mg being the most common. Most of the data came from adults with a single phase I pediatric trial of 27 patients with T2D aged 10–17 years who received a single dose of empagliflozin (5, 10, or 25 mg) [15].

The PK data were well described by a two-compartment model with sequential zero-first order absorption. Allometric scaling of all clearance and volume parameters provided an adequate description. The full covariate model provided an adequate description of the analysis data across dose groups and trials (adult and pediatric data [15]) with no overt biases across a wide range of covariates (age, weight, eGFR) via standard diagnostics and various simulation based pc-VPCs (data not shown). Further details of the final model appear in Supplementary Materials, with final parameter estimates provided in Table S7.

#### Empagliflozin PK-PD Model

The empagliflozin PK-PD model dataset included 6683 participants from 10 trials (Table S1) with a total of 45,541 PD observations. Doses in these trials ranged from 1 − 50 mg.

The PK-PD model for empagliflozin exposure on HbA1c was modeled using an indirect response model linked to AUC through an *I*_max_ model. Model diagnostics showed that the model described the observed data well. Further information on the model appears in Supplementary Materials, with final parameter estimates provided in Table S8.

The full covariate PK-PD model provided an adequate description of the analysis data via standard diagnostics and various simulation-based VPCs of longitudinal HbA1c changes across dosing regimens, trials, and relevant covariates. A specific emphasis was placed upon stratifications by baseline HbA1c, body weight, eGFR, and background medication.

#### Linagliptin PopPK Model

The linagliptin PopPK analysis-model dataset included 481 participants from 5 trials [16, 25–28] (TableS2), with 7005 linagliptin concentration observations, of which ~ 0.4% were below the LLOQ The doses in the analysis-model dataset ranged from 0.5 − 10 mg, mostly coming from the 2.5 − 10 mg range. The EBE-model dataset (mostly from the 5 mg dose group) included 1955 participants from 8 trials [16, 25–31] with 9493 observations; 0.4% being below the LLOQ.

The updated model included a single saturable binding component in the central compartment to account for the non-linear PK of linagliptin. This model is identical to well-established models for drugs that exhibit target-mediated drug disposition [41, 42] where linagliptin can undergo saturable binding to its target (DPP-4 activity).

Further information on the model appears in Supplementary Materials, with final parameter estimates in Table S9.

GOF plots showed that the model was able to describe the data well, including data from a pediatric trial [16] (data not shown). In simulation-based VPCs, the model was generally consistent with observed data in the central tendency across doses and trials (data not shown). Diagnostic plots for the validation EBE-model dataset also showed a reasonable fit.

#### Linagliptin PK-PD Model

The linagliptin population PK-PD model included 2964 participants with 14,352 HbA1c observations (Table S2). In addition to the placebo treatment group, the linagliptin doses available in the analysis dataset ranged from 0.5 − 10 mg, with the majority coming from 5 mg dose groups in adults (linagliptin 1 and 5 mg doses were evaluated in a pediatric trial [16]).

HbA1c from the time of first dose to 24 or 28 weeks was modeled using a turnover exposure-response model where HbA1c was produced at a zero-order rate (k_in_) and consumed at first-order process (k_out_). Linagliptin was assumed to inhibit HbA1c production by an inhibitory E_max_ model, with *I*_max_ and linagliptin AUC_ss_ producing AUC_50_ estimated from the data. The covariate model included assessments for placebo, wash-out of prior antidiabetic therapy before randomization, and impact of metformin and insulin co-therapy. Further information on the model appears in Supplementary Materials, with final parameter estimates in Table S10.

Simulation based VPCs of HbA1c versus time showed that longitudinal HbA1c simulated from the final model were similar to the observed data in the analysis set across dose groups, trials, and in pediatric and adult participants with T2D; adequate performance was also achieved when stratifying by background medication (data not shown).

### Bayesian Borrowing Based on Exposure-Response Data

#### Empagliflozin

The estimated mean (SE) placebo-corrected treatment effect from DINAMO was − 0.84% ± 0.33%, from which the likelihood was derived (Table 1). Population simulations based on previously-fitted pharmacometric models resulted in a population mean treatment effect estimate for empagliflozin of − 1.02%, with an SD of the distribution of the treatment effect of 0.06% (Figure S1A).

**Table 1:** Bayesian analysis of change in HbA1c (%) based on exposure-response data for empagliflozin (A) and linagliptin (B)

Parameter	Mean (SD)	Quantile							Probability superiority	ESS design^b^	ESS post-hoc^c^
		2.5%	5%	10%	50%^a^	90%	95%	97.5%			
**(A) Empagliflozin**											
Prior (exposure-response based)^d^	‒1.02 ± 1.37	‒4.38	‒3.47	‒2.32	‒1.02	0.27	1.42	2.33	0.89	51	55
Likelihood (DINAMO data)^e^	‒0.84 ± 0.33	‒1.50	‒	‒	‒	‒	‒	‒0.19	‒	‒	‒
Posterior distribution	‒0.95 ± 0.21	‒1.35	‒1.28	‒1.21	‒0.96	‒0.69	‒0.61	‒0.53	> 0.99	128	138
**(B) Linagliptin**											
Prior (exposure-response based)	‒0.64 ± 1.42	‒4.12	‒3.18	‒1.98	‒0.64	0.71	1.91	2.85	0.86	51	51
Likelihood (DINAMO data)^f^	‒0.34 ± 0.33	‒0.99	‒	‒	‒	‒	‒	0.30	‒	‒	‒
Posterior distribution	‒0.51 ± 0.22	‒0.92	‒0.85	‒0.78	‒0.52	‒0.25	‒0.15	‒0.05	0.98	128	128

CDI, credible interval: CI, confidence interval: ELIR, expected local information ratio: ESS, effective sample size: SE, standard error of the mean. Values shown are the placebo-corrected treatment effect of the change in HbA1c (%)

^a^Median

^b^ESS ELIR with design assumption about unit information standard deviation

^c^ESS ELIR post hoc based on observed data variability

^d^Based on 5,000 trial simulations of 158 patients each, with covariate values resampled from DINAMO

^e^From DINAMO primary analysis showing adjusted mean ± SE and 95% CI (*p* = 0.012)

^f^From DINAMO primary analysis, adjusted mean ± SE and 95% CI (*p* = 0.29)

The prior SD from the pharmacometric simulations (0.06) was less than the threshold for an ESS of 100 (0.229 = 2.29*/√*100). Therefore, the SD of the informative component was set to 0.229 to respect the ESS constraint. This resulted in a robust prior distribution described by the following equation (see Supplementary Materials for details of the equation):

$$\:{p}_{E}\left({\theta\:}_{E}\right)=0.65\:Norm\left(-1.02{,0.229}^{2}\right)+0.35\:Norm\left(-1.02,{2.29}^{2}\right)$$

The mean ± SD of this robust prior distribution was − 1.02 ± 1.37% (Table 1). Thus, after adjusting for relevant covariates, the predicted treatment effect for empagliflozin (− 1.02%) was similar to the observed treatment effect in DINAMO (− 0.84%).

**Fig. 2:**
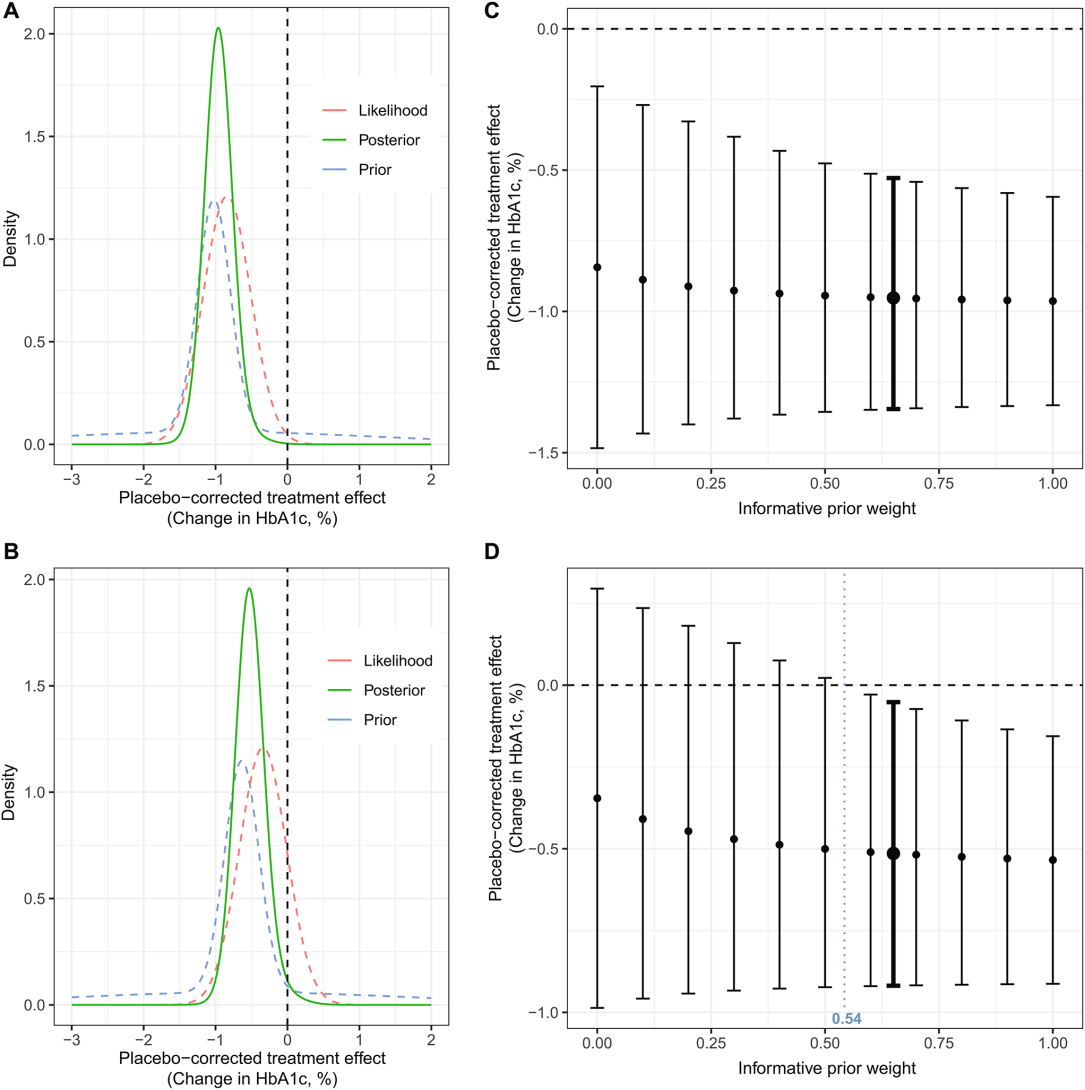
Bayesian analysis simulation results based on exposure-response placebo-corrected treatment effect distribution data for empagliflozin (**A**) and linagliptin (**B**); and tipping point sensitivity analysis of empagliflozin (**C**) and linagliptin (**D**) placebo-corrected treatment effect (change in HbA1c, %) and 95% credible interval (CDI) for different weights (range 0 to 1) of the informative prior. In **C** and **D**, a prior weight for the informative prior component of 0 corresponds to using only the weakly-informative prior and 1 corresponds to using only the pharmacometric simulation results as the prior; the bold interval corresponds to the pre-specified informative weight of 0.65 used in the primary analysis. The horizontal dashed line corresponds to the null effect value (0). The vertical intervals are 95% credible intervals; the points are the posterior mean

Comparison of the prior, likelihood, and posterior distributions of the mean placebo-corrected treatment effect did not show any evidence of prior-data conflict (Fig. 2A). The prior and likelihood distributions substantially overlapped, so the posterior had lower variance than using just the likelihood (i.e., a frequentist analysis).

Prior distributions were then updated with DINAMO data to obtain posterior distributions. The posterior mean placebo-corrected treatment effect was − 0.95%, with a SD of 0.21% (Table 1). The 97.5% quantile was − 0.53%, which was less than 0 corresponding to superior efficacy for empagliflozin compared with placebo. The posterior probability of the placebo-corrected treatment effect being less than 0 was greater than 0.99. The tipping point sensitivity analyses showed that for any choice of prior mixture weight, the 97.5% decision threshold was satisfied (Fig. 2C). Furthermore, as the informative prior weight increased (from 0 to 1), the width of the CDIs decreased, and the mean estimate became closer to the prior mean (‒1.02%). This reflected the increased information and lower variability in the informative prior compared with the robust prior component.

#### Linagliptin

The estimated mean (± SE) placebo-corrected treatment effect from the DINAMO trial was − 0.34% ± 0.33%, from which the likelihood was derived (Table 1).

Population simulations based on the previously-fitted pharmacometric models resulted in a population mean ± SD treatment effect estimate for linagliptin of − 0.64 ± 0.02% (Figure S1B). Prior distributions were updated with DINAMO data to obtain posterior distributions. Like with empagliflozin, the prior SD from the pharmacometric simulations (0.02) was less than the threshold for an ESS of 100 (0.238 = 2.38*/√*100). Therefore, the SD of the informative component was set to 0.238 to respect the ESS constraint. This resulted in a robust prior distribution described by the following equation (see Supplementary Materials for details of the equation):

$$\:{p}_{L}\left({\theta\:}_{L}\right)=0.65\:Norm\left(-0.64{,0.238}^{2}\right)+0.35\:Norm\left(-0.64{,2.38}^{2}\right)$$

The resulting robust prior distribution had a mean of − 0.64% and SD of 1.42% (Table 1). Thus, after adjusting for relevant covariates, the predicted treatment effect for linagliptin (− 0.64%) was greater than that observed in DINAMO (− 0.34%) (Fig. 2B). The prior mean deviated from the observed DINAMO mean (likelihood estimate) by 1 SE (Fig. 2B); however, this was insufficient evidence of prior-data conflict to substantially down-weight the prior, with substantial overlap of distributions.

The posterior mean placebo-corrected treatment effect was − 0.51% with an SD of 0.22% (Table 1). The 97.5% quantile was − 0.05%, which was less than 0 corresponding to superior efficacy for linagliptin versus placebo. The posterior probability of a placebo-corrected treatment effect less than 0 was 0.98.

Although the Bayesian analysis that borrowed information from pharmacometric simulations supported superior efficacy of linagliptin versus placebo, the result from this analysis was sensitive to the prior weight assigned to the informative prior. Importantly, the weight of 0.65 used in the primary analysis leading to superior efficacy was pre-specified and developed based on input from clinical experts and the FDA, so the choice of the included weight was not dependent on the observed DINAMO results. With an informative prior weight of 0, none of the three potential decision rules (97.5%, 95%, 90% quantiles) were met, consistent with the DINAMO trial results that did not confirm superior efficacy of linagliptin versus placebo. At an informative weight of 0.5, the 90% and 95% decision rules were met.

The tipping point sensitivity analyses showed that an informative prior weight of 0.542 resulted in 97.5% posterior probability of the placebo-corrected treatment effect being less than 0 (Fig. 2D). Thus, the prespecified criterion for superiority of linagliptin compared with placebo would not have been met with any choice of prior weight smaller than 0.542. This indicated substantial borrowing of adult data was required to establish superiority of linagliptin in the pharmacometrics-based model.

## Discussion

This Bayesian borrowing analysis combined prior data from pharmacometric simulations and results from DINAMO to increase power of the analysis to detect placebo-corrected treatment effects in a pediatric population. Empagliflozin and linagliptin have been extensively studied in adult populations, and the well-developed PK-PD models reported here supported this inference in pediatrics.

The use of PEBB in this analysis combined advantages of mechanistic modeling of differences between adults and young people with T2D, with advantages of partial extrapolation through Bayesian dynamic borrowing. A transparent quantitative approach provided a way to aggregate knowledge about efficacy of empagliflozin and linagliptin in adults, the recognized limited data in children and adolescents, and assumptions about relevance of adult data for pediatric efficacy. These findings supported the robustness of the DINAMO trial results and increased confidence in their validity.

Confidence in reliability of using the developed PopPK and PK-PD models to extrapolate adult data into a pediatric population relied on three factors. First, PK for each drug was assumed to be reasonably extended to pediatric populations after accounting for relevant covariate adjustments (e.g., weight, renal function, sex, race, etc.). Secondly, similarity in response to treatment was supported by analyses of short-term markers of efficacy (UGE for empagliflozin and DPP-4 inhibition for linagliptin), which were observed to be similar in pediatric and adult patients with T2D. Accordingly, extrapolation of exposure-response relationships were assumed to be valid when controlling for renal function, baseline HbA1c, and background medications (without direct adjustment for age as an independent covariate). Lastly, the potential impact of differences in disease progression between adults and pediatric patients on placebo-corrected responses were assessed via simulation (Boehringer Ingelheim, data on file). Compared with developed PK-PD models without disease progression, sensitivity analyses that incorporated differences in disease progression resulted in increases in the predicted placebo-adjusted changes in HbA1c. It follows that predictions based on the current models (which do not account for any potential differences in disease progression), result in conservative predictions of placebo-adjusted changes from baseline. Taken together, the assumed covariate-adjusted similarity in PK and PD, along with characterization of potential biases due to differences in disease progression, justifies the application of models for pediatric extrapolation of placebo-adjusted HbA1c.

The analysis described here highlights several important findings. First, the inclusion of a robust prior in the Bayesian model considered the possibility that pharmacometric simulations in adults might not be exchangeable with the pediatric population. As a result, the ability of the analysis methodology to detect prior-data conflict increased the confidence in the conclusions from DINAMO without any undue or excessive influence from the prior. Robust parametric approximations have been proposed and previously used in pediatric partial extrapolation settings [7, 40]. In this instance, the prior and the data had shown largely overlapping distributions of effect size. Using established PK-PD models to develop the prior distribution helped to leverage specific model-informed pre-existing knowledge about empagliflozin and linagliptin.

For empagliflozin, evidence of superior efficacy was seen across all decision rules and sensitivity analyses and was evident without use of Bayesian borrowing. DINAMO results showed that an empagliflozin dosing regimen provided clinically and statistically meaningful reductions in HbA1c in young people with T2D. Bayesian borrowing analysis similarly confirmed evidence for clinically meaningful efficacy of empagliflozin. Due to information gain, estimated CDIs of the treatment effect in the Bayesian borrowing analysis were narrower than the CI for the traditional analysis using only DINAMO data.

For linagliptin, predicted treatment effect was greater than that observed in DINAMO. The analysis using mixture prior distributions would have appropriately adjusted for prior-data conflict if substantial prior-data conflict had been observed. However, in the primary analysis, prior and likelihood were still centered in the same region, with the informative component of the prior distribution substantially overlapping the likelihood.

Combining previous knowledge with observed data in DINAMO substantially decreased variability of the linagliptin placebo-corrected treatment effect estimate. In the linagliptin analysis, HbA1c treatment effect point estimate shifted by ‒0.17% (from ‒0.34% to ‒0.51%), and the upper limit of the 95% CDI decreased by ‒0.35% (from + 0.30% to ‒0.05%) compared with observed DINAMO results. The reduction in variability and the stronger effect in the prior both contributed to the positive outcome. From the tipping point sensitivity analysis, it could be seen that choice of the prior distribution had a consequential effect on the strength of evidence associated with determining superior efficacy.

The primary trial analysis assumed no prior knowledge about magnitude and direction of the treatment effect. However, given previous trials showing superior efficacy of linagliptin in adult populations, epistemic equipoise (uncertainty) in the pediatric population is inconsistent with the totality of historical evidence. The failure to demonstrate superiority of linagliptin to placebo in primary trial analysis is likely due to inadequate efficacy, in combination with increased variability.

Although Bayesian estimation of nonlinear mixed effects models is a well-recognized analysis option in pharmacometric models [43], there are few examples where models have built priors of trial outcomes. In one such example, Sebastien and colleagues described a model bridging a biomarker with clinical endpoints to build a prior for pediatric trials [12]. Their approach did not involve a robust mixture prior or dynamic borrowing. Besides this, our work illustrates how population PK-PD models can be used to derive robustified priors for subsequent treatment comparisons in potentially limited target populations.

## Conclusion

The use of PEBB in this analysis combined advantages of mechanistic modeling of pharmacometric differences between adults and young people with T2D, with advantages of partial extrapolation through Bayesian dynamic borrowing. The Bayesian borrowing analysis based on pharmacometric extrapolation provided supportive evidence for efficacy for both empagliflozin and linagliptin.

Our findings suggest that the described PEBB approach is promising for future pediatric trials to increase power by leveraging information from previous trials through pharmacometric modeling and simulation.

## Electronic Supplementary Material

Below is the link to the electronic supplementary material.

Supplementary Material 1

## Data Availability

No datasets were generated or analysed during the current study.
