# Supplementary material for: Pharmacometrics-Enhanced Bayesian Borrowing for Pediatric Extrapolation – A Case Study of the DINAMO Trial
Source: Ther Innov Regul Sci. 2024 Oct 7;59(1):112–23. doi: 10.1007/s43441-024-00707-5 (PMC11706882; doi:10.1007/s43441-024-00707-5)
Supplement: Supplementary file 1 — Supplementary Material 1 [file 43441_2024_707_MOESM1_ESM.docx]

# **SUPPLEMENTAL METHODS**

**1.1 Software for analyses**

The population pharmacokinetic (PopPK) and pharmacokinetic-pharmacodynamic (PK-PD) analyses reported here were performed using nonlinear mixed-effects modelling with NONMEM^®^ (version 7.4, ICON Development Solutions, Ellicott City, Maryland, USA). Population and individual model parameters for the two PopPK models (and the linagliptin PK-PD model) were estimated using the first-order conditional estimation method with *η*–*ε* interaction (FOCEI) during model development. Full Bayesian estimation was employed for the final empagliflozin PopPK model [1]. Simulations, post-processing, and Bayesian borrowing analysis were performed using R version 4.1.1; R version 4.1.2 was used for the calculation of operating characteristics (R Core Team, R Foundation for Statistical Computing, 2021) [2]. RBesT (R Bayesian evidence synthesis tools, version 1.6.3) was used for the Bayesian borrowing analysis [3].

## **1.2 Pharmacometric Models**

**1.2.1 PK Sampling and Bioanalytical Assays**

Blood sampling schema for measurement of empagliflozin and linagliptin plasma concentrations were collected at selected times throughout each trial (**Table S3**). Empagliflozin and linagliptin plasma concentrations were analyzed by validated liquid chromatography-mass spectrometry (HPLC-MS/MS) methods, as described previously [4, 5]. The lower limits of quantification (LLOQ) were 1.11 nmol/L for empagliflozin [4] and 0.106 nmol/L for linagliptin [6]. Concentrations below the LLOQ were omitted from the analyses.

### **1.2.2 Empagliflozin PopPK Model**

A two-compartment model with sequential zero-first order absorption and fixed allometric scaling of all clearance and volume parameters was fitted to the empagliflozin PK data. The final model included covariates on the apparent clearance after oral dosing (CL/F) as described by the equation:

$$\frac{CL}{F_{i}}= {WT}_{CL}\cdot{EGFR}_{CL}\cdot e^{\theta_{1}+ {AGE}_{CL}+{BLACK}_{CL}+{ASIAN}_{CL}+{FEMALE}_{CL}+ {\eta1}_{i}}$$

$${WT}_{CL}=\left( \frac{{WT}_{i}}{70} \right)^{\theta_{7}}$$

$${EGFR}_{CL}=\left( \frac{{EGFR}_{i}}{90} \right)^{\theta_{11}}$$

$${AGE}_{CL}=\theta_{12} \cdot ln\left( \frac{{AGE}_{i}}{55} \right)$$

$${BLACK}_{CL}=\theta_{13} \cdot{BLACK}_{i}$$

$${ASIAN}_{CL}=\theta_{14} \cdot{ASIAN}_{i}$$

$${FEMALE}_{CL}=\theta_{15} \cdot{FEMALE}_{i}$$

where:

- the individual *i* value for CL/F was described as a function of individual body weight, eGFR, age, race, and sex, normalized by the typical population reference value.
- *θ*_1_ is the estimate of the typical population CL/F
- *θ*_7_, *θ*_11_, *θ*_12_, *θ*_13_, *θ*_14_, and *θ*_15_ are the estimates of weight, eGFR, age, race, and gender on the typical population CL/F
- η1*_i_* is the estimate of interindividual variability of CL/F, for individual *i*
- *BLACK_i_, ASIAN_i_*, and *FEMALE_i_* are binary variables corresponding to subject race or sex (TRUE=1, FALSE=0)
- *EGFR_i_* is a truncated eGFR measure for individual *i*, in which all measures greater than 120 mL/min/1.73m^2^ are set to 120.

The remaining model parameters apparent central volume of distribution after oral dosing (V_c_/F), apparent peripheral volume of distribution after oral dosing (V_p_/F), and apparent (oral) intercompartmental clearance (Q/F) were parameterized only as a function of body weight using a standardized power model, like the relationship in CL/F. As with CL/F, the exponents for V_c_/F, V_p_/F, and Q/F were fixed to literature allometric values 1.0, 1.0, and 0.75, respectively.

### **1.2.3 Empagliflozin PK-PD Model**

The exposure-response structural model describes how increasing empagliflozin exposure (area under the concentration-time curve at steady state [*AUC_ss_*]) stimulates a decrease in HbA1c levels. The final model is structured by the equations:

$$\frac{dHbA1c}{dt}=KIN \cdot\left( 1- {INH}_{i} \right)-HbA1c \cdot KOUT \cdot\left( 1+MPAST1 \cdot PLAC \right)$$

$${INH}_{i}= \frac{IMAX \cdot{AUC}_{ss,i}}{AUC50 \cdot{AUC}_{ss,i}}$$

$$KOUT= e^{\theta_{1}+ {\eta1}_{i}}$$

$${KIN}_{i}={KOUT}_{i} \cdot{BASE}_{i}$$

where:

- *KIN* is the zero order HbA1c synthesis rate.
- *MPAST*1 is a binary indicator for if time is greater than 0.
- *KOUT* is the first order HbA1c degradation rate constant.
- *PLAC* is the placebo response parameter.
- *IMAX* is the maximal inhibition.
- *AUC*50 is the empagliflozin AUC at which half the maximal effect is achieved.
- *AUC_ss_* is the subject-level empagliflozin area under the concentration time curve at steady state derived from the final population PK model.

Population and individual model parameters in NONMEM were estimated using the stochastic approximation expectation maximization (SAEM) estimation method [1]. A total of 10 iterations of importance sampling (expectation only) were used to assess the objective function value (OFV) and covariance matrix.

Covariates on baseline HbA1c were included in the final model, as described by the equation:

$${BASE}_{i}= e^{\theta_{2}+ {MET}_{BASE} + {INS}_{BASE} + {SULF}_{BASE} + {FEMALE}_{BASE} + {BLACK}_{BASE} + {ASIAN}_{BASE} + {AGE}_{BASE} + {WT}_{BASE} + {eGFR}_{BASE} + {\eta2}_{i}}$$

$${MET}_{BASE}= {MET}_{i}\cdot\theta_{6}$$

$${INS}_{BASE}= {INS}_{i}\cdot\theta_{7}$$

$${SULF}_{BASE}= {SULF}_{i}\cdot\theta_{8}$$

$${FEMALE}_{BASE}= {FEMALE}_{i}\cdot\theta_{12}$$

$${BLACK}_{BASE}= {BLACK}_{i}\cdot\theta_{15}$$

$${ASIAN}_{BASE}= {ASIAN}_{i}\cdot\theta_{16}$$

$${AGE}_{BASE}= \theta_{13} \cdot ln(\frac{{AGE}_{i}}{55})$$

$${WT}_{BASE}= \theta_{14} \cdot ln(\frac{{WT}_{i}}{70})$$

$${eGFR}_{BASE}= \theta_{17} \cdot ln(\frac{{eGFR}_{i}}{90})$$

where:

- the individual *i* value for baseline HbA1c (*BASE*) was described as a function of individual concomitant metformin, insulin and sulfonylurea, sex, race, age, body weight, and eGFR, normalized to the population reference value.
- *θ*_2_ is the estimate of the typical population baseline HbA1c.
- η1*_i_* is the estimate of interindividual variability of baseline KOUT, for individual *i.*
- η2*_i_* is the estimate of interindividual variability of baseline HbA1c, for individual *i.*
- *MET_i_*, INS*_i_*, and SULF*_i_* are binary variables corresponding to concomitant metformin, insulin, and sulfonylurea (TRUE=1, FALSE=0)
- *BLACKi*, *ASIANi*, and *FEMALE_i_* are binary variables corresponding to subject race or sex (TRUE=1, FALSE=0)
- *EGFR_i_* is a truncated eGFR measure for individual *i*, in which all measures greater than 120 mL/min/1.73m^2^ are set to 120.

Covariates on *IMAX* were included in the full model as described by the equation:

$${IMAX}_{i}= \theta_{4} \cdot{eGFR}_{IMAX} \cdot{BASE}_{IMAX}$$

$${eGFR}_{IMAX}= \left( \frac{{eGFR}_{i}}{90} \right)^{\theta_{18}}$$

$${BASE}_{IMAX}= \left( \frac{{BASE}_{i}}{90} \right)^{\theta_{19}}$$

where:

- the individual *i* value for IMAX was described as a function of individual baseline HbA1c and eGFR, normalized to the population reference value.
- *θ*_4_ is the estimate of the typical population maximal inhibition.
- eGFR*i* is a truncated eGFR measure for individual *i*, in which all measures greater than 120 mL/min/1.73m^2^ are set to 120.
- BASE*i* is the individual observed baseline HbA1c.

Covariates on *PLAC* were included in the full model as described by the equation:

$${PLAC}_{i}= e^{\theta_{3}+ {MET}_{PLAC} + {INS}_{PLAC}+ {SULF}_{PLAC}+ {\eta3}_{i}}$$

$${MET}_{PLAC}= {MET}_{i} \cdot\theta_{9}$$

$${INS}_{PLAC}= {INS}_{i} \cdot\theta_{10}$$

$${SULF}_{PLAC}= {SULF}_{i} \cdot\theta_{11}$$

where:

- the individual *i* value for PLAC was described as a function of concomitant metformin, insulin, and sulfonylurea.
- *θ*_3_ is the estimate of the typical population placebo response.
- η3*_i_* is the estimate of interindividual variability of placebo response, for individual *i*.
- *MET_i_*, *INS_i_*, and *SULF_i_* are binary variables corresponding to concomitant metformin, insulin, and sulfonylurea (TRUE=1, FALSE=0).

### **1.2.4 Linagliptin PopPK Model**

Due to high variability in linagliptin concentrations in the phase 3 trials, a three-step modelling approach was used. First, a base model was developed using data from 4 early to mid-phase adult trials [7-10] and a pediatric trial [11] (described here as the analysis-model dataset). These trials provided densely sampled data across a wide range of doses to characterize the structural model and to obtain a reasonable description of the variability in the non-linear PK present in the data, including differences in adult and pediatric PK. The previously developed two-compartment PK model with first order absorption and saturable protein binding in the central and peripheral compartments was used as a starting point [12]. Alternative parameterizations to improve stability and/or interpretability were explored, as needed, with the goal of establishing a base model that provided unbiased predictions for both adult and pediatric patients. Covariates evaluated included those of scientific interest in a previous pediatric model analysis [13] along with weight, age, estimated glomerular filtration rate [eGFR], DPP-4 activity, patient sex, and race. Weight was used to scale PK model clearances and volumes with fixed allometric exponents (0.75 for clearance and 1.00 for volume) due to potential differences in weight for pediatric patients. Age and eGFR were incorporated on clearance (CL).

The final PopPK model for linagliptin consisted of a two-compartment model, parameterized in terms of CL/F, V_c_/F, V_p_/F, Q/F, and absorption rate constant (K_A_). All clearance and volume parameters were allometrically scaled with fixed coefficients of 0.75 and 1.0, respectively. A saturable binding component, with no target-mediated elimination, was added to the central compartment to account for the non-linear PK of linagliptin. The saturable binding component included estimable parameters for binding affinity and for total DPP-4 concentration (RMAX). The final base model included IIV on CL/F and K_A_ in a diagonal matrix. The residual error model consisted of two separate log-additive error terms with one for the two phase I trials [7, 8] and one for the non-Phase I trials (all remaining trials). To help account for potential variability due to occasion (PK sampling visit), inter-occasion variability (IOV) was included on relative bioavailability in oral absorption across occasions within subjects.

Covariates included in the final model were described by the following equations:

$$\frac{{CL}_{i}}{F_{occ}}= \theta_{1} \cdot({\frac{{WT}_{i}}{80})}^{0.75} \cdot{\theta_{11}}^{SEX\left[ Female \right]} \cdot e^{\eta_{\frac{CL}{F}}}$$

$$\frac{{V2}_{i}}{F_{occ}}= \theta_{2} \cdot({\frac{{WT}_{i}}{80})}^{1.0}$$

$$\frac{Q_{i}}{F_{occ}}= \theta_{3} \cdot({\frac{{WT}_{i}}{80})}^{0.75}$$

$$\frac{{V3}_{i}}{F_{occ}}= \theta_{4} \cdot({\frac{{WT}_{i}}{80})}^{1.0}$$

$$F_{occ}= e^{{\eta IOV}_{occ}}$$

$$\mathrm{KA}= \theta_{6} \cdot e^{\eta_{ka}}$$

$$\mathrm{KSS}= \theta_{7}$$

$$\mathrm{RMAX}= \theta_{8}$$

$$C_{total} =\frac{{AMT}_{CMT2}}{\frac{V2}{F_{occ}}}$$

$$C_{free}=0.5 \cdot\left( C_{total}-\mathrm{RMAX}-\mathrm{KSS} \right)+0.5 \cdot\sqrt{{(C_{total}-RMAX-KSS)}^{2}+4 \cdot KSS \cdot C_{total}}$$

where:

- individual PK parameters are denoted by the subscript *i*, and occasion specific parameters are denoted by subscript *occ*.

### **1.2.5 Linagliptin PK-PD Model**

Using the maximum a posteriori (MAP) predictions from the final PK modelling step, AUC_ss_ were generated for each subject in the longitudinal HbA1c data set, including all subjects with at least one baseline and one post-baseline HbA1c measurement from 8 trials included in the EBE-model dataset [7-11, 14-16].

The exposure-response relationships between HbA1c and linagliptin exposure (AUC_ss_) were characterized using a turnover model for HbA1c, as described below:

$$\frac{dHbA1c}{dt}=KIN \cdot\left( 1- {INH}_{i} \right)-HbA1c \cdot KOUT+WOEFF$$

$${INH}_{i}= \frac{IMAX \cdot{AUC}_{ss,i}}{AUC50 \cdot{AUC}_{ss,i}}$$

where:

- *HbA*1*c* is the repeated measured (within subject) HbA1c.
- *KIN* is the zero-order HbA1c synthesis rate.
- *KOUT* is the first-order HbA1c degradation rate constant.
- *IMAX* is the maximal inhibition.
- *AUC50* is the linagliptin *AUC*_ss_ at which half the maximal effect is achieved.
- *AUC_ss_* is the subject-level linagliptin area under the concentration time curve at steady state derived from the final population PK model.

A physiologic lower limit on HbA1c was evaluated to maintain ranges in the model within physiologically plausible limits. The limitation was implemented as a fractional change in *KOUT*. This approach has been used previously in models of HbA1c [17-19].

Population and individual model parameters were estimated using the first-order conditional estimation with eta-epsilon interaction estimation method for both the base and final model.

Metformin and insulin were modelled as simple time-invariant effects on both the baseline HbA1c value and the zero-order production of HbA1c.

Covariates included in the final model were described by the following equations:

$${BASE}_{i}= e^{\theta_{1}+ {MET}_{i} \cdot\theta_{8}+ {INS}_{i} \cdot\theta_{9}+ \eta_{1}}$$

$${KIN}_{i}= e^{\theta_{2}+ {MET}_{i} \cdot\theta_{4}+ {INS}_{i} \cdot\theta_{5}}$$

$${WOEFF}_{i}= e^{\theta_{3}} \cdot{Washout}_{i}$$

$$KOUT= \frac{KIN}{BASE}$$

$$IMAX=\frac{e^{\theta_{6}}}{\left( 1+ e^{\theta_{6}} \right)}$$

$$AUC50= e^{\theta_{7}}$$

where:

- Wash−out*_i_* is the individual indicator for wash-out of previous antidiabetic medication
- WOEFF is the zero-order input representing the effect of wash-out of previous antidiabetic medication
- *MET_i_* and *INS_i_* are indicators for metformin and insulin background therapy in the *i*th individual, respectively.
- *θ_1_* is HbA1c at the start of therapy.
- *θ_3_, θ_4_* and *θ_5_* are estimable parameters describing impact of wash-out, metformin, and insulin on HbA1c dynamics, respectively.
- IMAX is maximum inhibitory effect of linagliptin.
- *AUC*50 is linagliptin AUCss for half-maximal inhibition.

### **1.2.6 Model Selection and Evaluation**

Model selection for the pharmacometric models was guided by numerical change in objective function values (OFV), physiological relevance of parameter estimates, precision of parameter estimates, minimization of Akaike information criterion, and overall GOF plots. GOF plots included diagnostic scatter plots, figures of normal prediction distribution error (NPDE) values versus time and population predicted values, and plots of observed concentrations versus population and individual predicted concentrations. Models run using the Bayesian estimation method were analyzed for convergence using density plots of posterior parameter distributions.

For the longitudinal VPCs, parameter uncertainty was assumed negligible relative to inter-individual and residual variance [20]. VPCs were performed with Monte-Carlo simulations. Five hundred datasets, identical in structure to the original dataset, were simulated using the developed PopPK models. Plots of the observed empagliflozin and linagliptin plasma concentrations were then constructed and the observed median, 5th, and 95th percentiles were overlaid with the corresponding 95% prediction interval at each percentile for the simulated concentrations over time.

### **1.2.7 Model Simulations**

Simulations for the pharmacometric models were conducted in an iterative manner for a total of 5,000 iterations with one iteration consisting of the following key steps:

Using blinded demographic and background medication data from DINAMO (158 randomized participants), 10,000 individual participants were resampled without stratification, with 5,000 per treatment group (linagliptin and empagliflozin).

AUC_ss_ values were generated for each active treatment patient for linagliptin 5 mg and empagliflozin 10 mg and 25 mg (anticipating potential need for re-randomization), respectively. Covariates were sampled jointly, on a per-subject basis which ensured a realistic multivariate structure.

- A typical value of CL/F and its associated inter-individual variance was generated by sampling from the respective uncertainty distributions (Bayesian posterior for empagliflozin; non-parametric bootstrap for linagliptin). A typical value for inter-occasion variability in absolute bioavailability (F) was also generated for linagliptin.
- Individual level CL/F parameters were generated from a normal distribution with a mean of zero and variance set to the sampled population variance described in the preceding step.
- For the linagliptin model, an occasion specific F was generated from a normal distribution with a mean of zero and variance set to the sampled population IOV described in the preceding step.
- For empagliflozin, AUC_ss_ was calculated by dividing the administered dose via the individual CL/F. For linagliptin, the AUC_ss_ was derived by integrating over the individual concentration time profile for an interval of 24 hours under steady-state conditions.

Using the AUC_ss_ values, longitudinal HbA1c were simulated for each patient, out to 26 weeks.

- Typical values, inter-individual variances, and residual error variances were generated by sampling from an uncertainty distribution for the respective model (asymptotic standard errors for empagliflozin; non-parametric bootstrap for linagliptin as these were the methods used for assessing parameter uncertainty).
- A sample for individual level parameters was generated from normal distributions with means of zero and variances set to the sampled population variance described in the preceding step.
- HbA1c observations were simulated at baseline, 12 weeks, and 26 weeks. For participants assigned to the empagliflozin treatment arm, a re-randomization will be performed at week 12 for participants not achieving HbA1c values <7.0%.
- To derive the placebo corrected response for simulated patients, their respective counterfactual placebo response was simulated, and the placebo adjustment derived accordingly.

The mean and standard deviation (SD) of the placebo-corrected HbA1c change from baseline was then calculated for each iteration and treatment group.

## **1.3 Bayesian Borrowing Based on Exposure-Response**

**1.3.1 Prior Calculation**

**Pharmacometric model predictions**

For both comparisons of interest (i.e. placebo-corrected HbA1c change from baseline for empagliflozin and linagliptin), the sample means from each of the 5,000 corresponding iterations in the pharmacometric simulation constitute a random sample of the predicted placebo-corrected treatment effect in DINAMO. Due to the nature of the pharmacometric models, it was assumed that the predicted placebo-corrected mean HbA1c change from baseline approximately follows a normal distribution (**Figure S1**). For each comparison separately, the mean $\mu_{I}$ of this distribution was estimated by the arithmetic mean of the 5000-sample means. The variance $v_{I}$ of this distribution was also estimated by the sample variance of the 5000-sample means.

**Prior ESS calculation**

To calculate the prior effective sample size (ESS), the SD of the data was derived from clinical trials in adults and scaled for the pediatric population based on differences between the blinded assessment of the DINAMO trial results and the historical adult clinical trials.

The unit-information SDs $\sigma_{I}$ for the placebo-corrected treatment effects were initially estimated [21]. Here, the unit-information SD is the difference between one observation of HbA1c change from baseline with active treatment and one observation with placebo treatment in DINAMO. Since DINAMO was blinded at the time of this analysis, the DINAMO data was not used directly. Instead, clinical trial results from adults were used to estimate the unit-information SD per treatment group. For the HbA1c change with empagliflozin, results from 3 trials [22-24] were used. For linagliptin, results from 2 trials [16, 25] were used. For placebo, the treatment results from all 5 trials [16, 22-25] were used. The unit-information SD for one observational unit was estimated separately by treatment group, as the pooled SD given by:

$s=\sqrt{(\sum\left( N_{i}-1 \right)s_{i}^{2})/\sum\left( N_{i}-1 \right)}$,

where:

$N_{i}$ is the sample size and $s_{i}$ is the SD in study $i$.

Here, $s_{i}$ were 0.81, 0.87, and 0.91 for empagliflozin, linagliptin, and placebo, respectively. The mean HbA1c change per treatment group based on a MAP prior analysis [3, 26] of the historical data was then estimated. Moderate prior between-study heterogeneity was assumed, modelling this as $\tau\sim HalfNormal\left( scale=\frac{\sigma}{8} \right)$ [21]. The MAP prior means based on the MAP analysis were calculated as −0.82, −0.66, and −0.17, for empagliflozin, linagliptin, and placebo, respectively.

It was assumed that the SD in the DINAMO treatment groups would be higher, and that the same scaling factor z would be used for the SD in each treatment group. Change from baseline data in DINAMO was simulated with mean estimates corresponding to the adult trials MAP prior estimates and SD corresponding to the scaled SD from adult trials. A scaling factor of z of 1.89 leads, on average, to a pooled SD in the DINAMO trial of 1.65. This was the pooled SD observed in the still blinded DINAMO trial. It was therefore assumed that the DINAMO SD would equal 1.52, 1.65, and 1.72, for empagliflozin, linagliptin, and placebo respectively. Consequently, the estimated unit-information SD for empagliflozin ($\sigma_{E}$) was calculated as 2.29 (= $\sqrt{{1.52}^{2} + {1.72}^{2}}$) and for linagliptin ($\sigma_{L}$) was 2.38 (= $\sqrt{{1.65}^{2} + {1.72}^{2}}$).

To obtain comparisons that correspond to an informative prior weight of at most 100 patients per treatment group, the prior variances $v_{I}$ from the pharmacometrics simulations were replaced with:

$$\upsilon_{I}^{*}=\left\{ \begin{aligned} v_{I}, if v_{I}\geq\sigma_{I}^{2}/100 \\ \frac{\sigma_{I}^{2}}{100}, else. \end{aligned} \right.$$

**Prior robustification**

The informative prior was then robustified against potential prior-data conflict. The final prior is a mixture of the informative prior part (weight $w$ of 0.65) and a weakly informative normally distributed prior component with mean $\mu_{I}$ corresponding to the mean placebo corrected treatment effect estimated from the pharmacometric simulation, and SD $\sigma_{I}$. From this, the final prior probability densities for the placebo corrected treatment effect ($\theta_{I}$) were:

$p_{I}\left( \theta_{I} \right)=w_{I}Norm\left( \mu_{I},\nu_{I}^{*} \right)+(1-w_{I})Norm\left( \mu_{I},\sigma_{I}^{2} \right)$,

where:

- *Norm* refers to the probability density function of the normal distribution.
- $\theta_{I}$ represents the placebo-corrected treatment effect (change in HbA1c, %).
- *I* is the treatment group of interest, i.e. empagliflozin or linagliptin.
- $w_{I}$ is the weight of the informative part of mixture prior (elicited with experts from the trial steering committee and 0.65 in this analysis; the FDA allowing for adjustment of this such that the prior ESS_ELIR_ [27] equals the planned sample size).
- $\mu_{I}$is the mean of the informative part of mixture prior (calculated as mean of 5000 means from PK-PD simulation for DINAMO population).
- $v_{I}^{*}$ is the variance of informative part of mixture prior calculated as sample variance of 5000 means from PK-PD simulation for DINAMO population; the lower limit for $v_{I}^{*}$ is specified such that informative part of prior corresponds to at most 100 patients per treatment group based on expert elicitation.
- $\sigma_{I}^{2}$is the variance of the robust part of mixture prior, based on unit-information SD $\sigma_{I}=2.29 \left( 2.38 \right)$ for empagliflozin (linagliptin) vs. placebo, with ESS_ELIR_ equal to 1 for the robust component.

**Weight of the informative prior**

Members of the DINAMO trial steering committee were also asked to assess the weight of the informative part of the prior relative to the robust weakly-informative part. Based on this advice, the weight for the informative part of the prior for the placebo-corrected treatment effect was initially set to 0.50. Here, a weight of 0 corresponded to a weakly-informative prior, with the resulting estimate being based almost entirely on the DINAMO data. A weight of 1 corresponded to a prior that is entirely based on the pharmacometric model predictions, assuming full exchangeability of the covariate-adjusted predictions with the DINAMO outcome data without robust component down-weighting. In subsequent correspondence with the FDA, it was recommended that the maximum number of effective borrowed patients as determined by the calculated ESS of the prior distribution should be no more than the total sample size of the DINAMO trial.

The prior distributions were summarized descriptively by mean, SD, 2.5%-, 5%-, 10%-, 90%-, 95%- and 97.5%-quantiles, quartiles including the median as well as the ESS. As there is no commonly agreed standard for calculating the ESS of a parametric mixture probability distribution, the expected local information ratio (ELIR) ESS, proposed by Neuenschwander and colleagues [27] was used as it satisfies the predictive consistency criterion, i.e. the ESS of the posterior will on average equal the ESS of the prior plus the sample size of the data provided the data is generated under the prior.

For reporting purposes, ESS ELIR was reported twice; both based on the planning assumptions and using the reference SD from the unblinded DINAMO results, which could not be used in the prior derivation.

**1.3.2 Posterior Calculation and Decision Rule**

The prior distribution is updated with the data *y_I_* from DINAMO using Bayes theorem. The primary analysis of covariance (ANCOVA) efficacy analysis in the DINAMO protocol was used to obtain estimated means $\hat{\mu}_{I}$ and variances $\hat{\upsilon_{I}^{2}}$ of the placebo-corrected treatment effects.

Corresponding normal distributions with densities *p_I_* (*y_I_* |*θ_I_*) were used to model the likelihood. The resulting posterior distributions were calculated by the equation:

$p_{I}\left( {\theta_{I}|y}_{I} \right)=\frac{{p_{I}(\theta_{I})p}_{I}\left( y_{I}|\theta_{I} \right)}{\int{p_{I}(\theta_{I})p}_{I}\left( y_{I}|\theta_{I} \right) d\theta_{I}}$.

RBesT was used to calculate the posterior distributions. The posterior distribution was summarized descriptively by mean, SD, 2.5%-, 5%-, 10%-, 90%-, 95%- and 97.5%- quantiles, quartiles including the median as well as the ESS. For reporting purposes, the ESS for the placebo-corrected treatment effects was expressed relative to the unit-information SD estimated from unblinded DINAMO results.

Plots of the prior density, likelihood, and posterior density for each treatment group were created for visual inspection of prior-data conflict.

The posterior distributions were used to describe and evaluate the placebo corrected treatment effects for empagliflozin and linagliptin, respectively. For each treatment, the decision rule in this analysis compared the 97.5% quantile of the posterior placebo-corrected treatment effect of empagliflozin (linagliptin) with 0 to make a statement whether there is at least a 97.5% probability that the placebo-corrected treatment effect of empagliflozin (linagliptin) is below 0. The criterion is therefore:

$$Prob\left( \theta_{I}<0 | y \right)\geq0.975$$

where:

- *y* is the observed data
- *θ_I_* is the placebo-corrected efficacy of empagliflozin (linagliptin)

If this criterion was met, then there was evidence of superior efficacy of empagliflozin (linagliptin) in the pediatric population of DINAMO. Lower values for *θ_I_* correspond to better efficacy of the active drug.

**1.3.3 Operating Characteristics**

During the planning of the supplementary Bayesian analysis, the operating characteristics (OC) (i.e. power and type I error) for the Bayesian analysis were calculated conditional on potential outcomes of the pharmacometrics simulations and corresponding prior derivation. The OC were calculated for a set of scenarios with varying assumptions about the true placebo-corrected treatment effect in the DINAMO population.

In addition, the OC were calculated for alternative choices of the informative prior part weight and ESS to assess the robustness of the choice of weight and ESS. The OC were calculated under the assumption that the unit-information SD for the placebo-corrected treatment effects in DINAMO were as assumed, i.e. corresponding to the observed pooled SD of 1.65.

The OC were calculated for evidence levels of 97.5% and 95% in the decision rule. For each scenario, the predictive probability of meeting the decision criterion was calculated with the oc1S function in RBesT [3]. A scenario with treatment superiority corresponded to a true positive decision. A scenario without treatment superiority was a false positive decision.

The corresponding OC meeting criterion for prior means for empagliflozin (*µ*_E_) and linagliptin (*µ*_L_) equal to −0.55 (mean differences in the HbA1c change from baseline to week 26 in the treatment groups versus placebo group) and equal to 0 (no treatment benefit) for an informative part ESS of 100 are shown in **Table S5** and **Table S6**.

Once the pharmacometrics simulations were completed, the final prior distribution for the analysis was known. At this point the type I error and power calculations can be updated (this updated calculation is referred to as conditional type I error rate [28]). **Figure S2** shows type I error and power as a function of the unit-information SD for the placebo-corrected treatment effects observed in the DINAMO trial. The maximum type I error is 18.2% (empagliflozin) and 20.5% (linagliptin) for the robust priors of each treatment if the SD in DINAMO differs from the SD assumed in the prior. If no prior robustification had been used, the maximum type I error would have been unbounded, i.e. have approached 100%.

**Figure S3** shows the potential impact on power due to potential bias introduced through the pharmacometric model simulations. Bias is defined as the prior mean minus the true placebo-corrected treatment effect in DINAMO. To illustrate a potential increase in power in a situation of marginal treatment benefit, a true placebo corrected mean of −0.20 is assumed. If the pharmacometric model simulations overestimate the treatment effect, the power would increase. Here, this potential increase is limited to 40.6% (empagliflozin) and 39.7% (linagliptin) for the robust prior while it is unbounded for non-robust borrowing.

# **SUPPLEMENTAL RESULTS**

**1.1 Empagliflozin results**

This robust prior distribution had a mean of −1.02% and an SD of 1.37% (**Table 1**). The 2.5% quantile (−4.38%) and 97.5% quantile (2.33%) were farther from the mean than the ~2 SDs as would have been expected in a normal prior; this was a result of the robustification of the prior.

### **1.2 Linagliptin results**

The robust prior distribution had a mean of −0.64% and an SD of 1.42% (**Table 1**). The 2.5% quantile (−4.12%) and 97.5% quantile (2.85%) were farther from the mean than the ~2 SDs as would have been expected in a normal prior; this was a result of the robustification of the prior.

**Table S1** Empagliflozin trials as data source for pharmacometric modelling

| **Trial** | **Reference** | **Clinical Phase** | **Population** | **Intervention** | **Comparator** | **Duration (weeks)** | **Endpoints** |
| --- | --- | --- | --- | --- | --- | --- | --- |
| 1245.2 | Heise 2013 [29] | I | T2D | EMPA 2.5, 10, 25, or 100 mg qd | PBO | 9 days | PK |
| 1245.4 | Heise 2013 [30] | I | T2D | EMPA 10, 25, or 100 mg qd | PBO | 4 | PK |
| 1245.9 | Ferrannini 2013 [31] | IIb | T2D | Met + EMPA 5, 10, 25 mg qd | PBO | 12 | PK; △HbA1c |
| 1245.10 | Rosenstock 2013 [32] | IIb | T2D not controlled on Met | Met + EMPA 1, 5, 10, 25, or 50 mg qd | Met + PBO, Met + sitagliptin 100 mg qd | 12 | PK; △HbA1c |
| 1245.15 | Kanada 2013 [33] | II | Japanese patients with T2D | EMPA 1, 5, 10, 25 mg qd | PBO | 4 | PK |
| 1245.19 | Kovacs 2014 [34] | III | T2D | Pioglitazone ± Met plus EMPA 10, 25 mg qd | Pioglitazone ± Met plus PBO | 24 | PK |
| 1245.20 | Roden 2013 [35] | III | Treatment naïve patients with T2D | EMPA 10, 25 mg qd | Sitagliptin 100 mg qd, PBO | 24 | PK; △HbA1c |
| 1245.23 | Haring 2013 [22] | III | T2D not controlled on Met plus SU | Met plus EMPA 10, or 25 mg qd | Met plus PBO | 24 | PK; △HbA1c |
| 1245.28 | Ridderstråle 2014 [36] | III | T2D not controlled on Met | Met + EMPA 25 mg qd | Met + SU | 104 | PK; △HbA1c |
| 1245.33 | Rosenstock 2015 [23] | IIb | T2D not controlled on basal insulin | Basala insulin plus EMPA 10, or 25 mg qd | Basal insulin plus PBO | 78 | PK; △HbA1c |
| 1245.36 | Barnett 2014 [37] | III | T2D with mild-severe renal impairment | Background ADA plus EMPA 10, 25 mg qd | Background ADA plus PBO | 52 | PK |
| 1245.87 | Laffel 2018 [38] | I | Single dose in adolescents 10–17 years with T2D | EMPA 5, 10, 25 mg qd | Nil | 1 day | PK |
| 1276.1 | Hadjadj 2016 [39] | III | T2D | Met + EMPA 5, 12.5 mg bid, 25 mg qd | Met | 24 | PK; △HbA1c |
| 1276.10 | NCT01649297 [40] | IIb | T2D with insufficient glycemic control | Met + EMPA 5, 12.5 mg bid, 10, 25 mg qd | Met + PBO | 16 | PK; △HbA1c |

bid, twice daily: EMPA, empagliflozin: HbA1c, glycated hemoglobin: LIN, linagliptin: MDI, multiple daily injections: Met, metformin: PBO, placebo: PK, pharmacokinetic: qd, once daily: T2D, type 2 diabetes: SU, sulfonylurea: △, change in.

**Table S2** Linagliptin trials as data source for pharmacometric modelling

| **Trial** | **Reference** | **Clinical Phase** | **Population** | **Intervention** | **Comparator** | **Duration (weeks)** | **Endpoints** |
| --- | --- | --- | --- | --- | --- | --- | --- |
| 1218.2 | NCT02183350 [7] | I | Males with T2D | LIN 1, 2.5, 5, 10 mg qd | PBO | 12 d | PK |
| 1218.3 | NCT02183415 [8] | I | Males with T2D | LIN 2.5, 5, 10 mg qd | PBO | 4 | PK; △HbA1c |
| 1218.5 | NCT00740051 [9] | IIb | T2D with insufficient glycemic control | LIN 0.5, 2.5, 5 mg qd | PBO | 12 | PK; △HbA1c |
| 1218.6 | NCT00309608 [10] | IIb | T2D not controlled on Met | Met + LIN 1, 5, 10 mg qd | PBO | 12 | PK; △HbA1c |
| 1218.16 | Del Prato 2011 [14] | III | T2D with insufficient glycemic control | LIN 5 mg qd | PBO | 24 | PK; △HbA1c |
| 1218.20 | Gallwitz 2012 [15] | III | T2D with insufficient glycemic control | LIN 5 mg qd | SU | 104 | PK; △HbA1c |
| 1218.36 | Yki-Järvinen 2013 [16] | III | T2D not controlled on basal insulin | Basal insulin + LIN 5 mg qd | Basal insulin | 52 | PK; △HbA1c |
| 1218.56 | NCT01342484 [11] | IIb | Children/adolescents aged 10-17 y with T2D | LIN 1, 5 mg qd | PBO | 12 | PK; △HbA1c |

bid, twice daily: HbA1c, glycated hemoglobin: LIN, linagliptin: Met, metformin: PBO, placebo: PK, pharmacokinetic: qd, once daily: T2D, type 2 diabetes: SU, sulfonylurea.

**Table S3** PK sampling in the included empagliflozin and linagliptin trials.

| **Trial** | **Reference** | **PK Sampling Design** |
| --- | --- | --- |
| **Empagliflozin** | | |
| 1245.2 | Heise 2013 [29] | Full PK Profiles (D1 and 9) plus daily troughs |
| 1245.4 | Heise 2013 [30] | Full PK Profiles (D1 and 28) plus daily troughs on D2-4, 7, 14, 21, 25, 26, 27, and 29 |
| 1245.9 | Ferrannini 2013 [31] | Pre-dose on D1, 28, 56, 84, plus 1-2 post-dose samples on D84 |
| 1245.10 | Rosenstock 2013 [32] | Pre-dose on D1, 28, 56, 84, plus 1-2 post-dose samples on D84 |
| 1245.15 | Kanada 2013 [33] | Full PK profiles (D1, D28), daily troughs on D2, 7 14, 21, 26, 27, 29, plus 12, 24, and 48 h after last dose |
| 1245.19 | Kovacs 2014 [34] | Trough samples on D85 and 169 |
| 1245.20 | Roden 2013 [35] | Trough samples on D85 and 169 plus two post-dose samples on D169 |
| 1245.23 | Haring 2013 [22] | Trough samples on D85 and 169 |
| 1245.28 | Ridderstråle 2014 [36] | Trough samples on weeks 12 and 28 plus two post-dose samples on week 28 |
| 1245.33 | Rosenstock 2015 [23] | Pre-dose on weeks 6, 12, 18, and two post-dose samples on week 18 |
| 1245.36 | Barnett 2014 [37] | Trough samples on D85 and 169 plus two post-dose samples on D169 |
| 1245.87 | Laffel 2018 [38] | Full PK profile for single dose |
| 1276.1 | Hadjadj 2016 [39] | Trough samples on weeks 12, 18, and 24 |
| 1276.10 | NCT01649297 [40] | Trough samples on weeks 4 and 16 |
| **Linagliptin** | | |
| 1218.2 | NCT02183350 [7] | Rich samples on D1/12; predose sample on D2 to 11; predose sample D13, 14, 16, 18, 20 |
| 1218.3 | NCT02183415 [8] | Rich samples on D1/28; predose on D2, 6, 12, 19 26 and 28; AM sample D29, 30, 33, 36, 39, 41, 43 |
| 1218.5 | NCT00740051 [9] | D1, 28, 56, 84, 98: predose sample, 1 and 2 hours post dose; visit 9: any time |
| 1218.6 | NCT00309608 [10] | D1, 28, 56, 84: predose, 1 and 2 hours post dose; D98 any time |
| 1218.16 | Del Prato 2011 [14] | Predose sample on D84 and 168 |
| 1218.20 | Gallwitz 2012 [15] | Predose sample, D196, 364 and 728 |
| 1218.36 | Yki-Järvinen 2013 [16] | Predose sample, D168 |
| 1218.56 | NCT01342484 [11] | Predose sample, D0 and 28; PK sub-study: rich samples on visit 4 (or alternatively visit 5 or 6) |

D, day: PK, pharmacokinetic.

**Table S4** Tested covariates for influence on PopPK profile and exposure-efficacy relationship of empagliflozin and linagliptin.

| **Covariate** | **Empagliflozin** | |  | **Linagliptin** | |
| --- | --- | --- | --- | --- | --- |
|  | **PopPK*** | **PK-PD** |  | **PopPK** | **PK-PD** |
| Age | ✓ | ✓ |  | ✓** |  |
| Body weight | ✓^#^ | ✓ |  | ✓^#^ |  |
| Race | ✓ | ✓ |  | ✓** |  |
| Sex | ✓ | ✓ |  | ✓ | ✓ |
| Estimated glomerular filtration rate (eGFR) | ✓ | ✓ |  | ✓** |  |
| Background metformin |  | ✓ |  |  | ✓ |
| Background insulin |  | ✓ |  |  | ✓ |
| Background sulfonylurea |  | ✓ |  |  |  |
| Baseline HbA1c |  | ✓ |  |  |  |
| DPP-4 activity |  |  |  | ✓ |  |

^#^Fixed allometrically for clearance and volume

*The covariates included on *CL/F*

**Not tested in initial model development due to unstable runs. Included in full covariate model using the full dataset EBE model.

**Table S5** Probability of meeting criterion for prior means for empagliflozin ($\mu_{E}$) and linagliptin ($\mu_{L}$) equal to −0.55 and informative part ESS of 100

| **Scenario**  **No.** | **Weight of**  **Informative part** | **True**  **treatment**  **effect in**  **children** | **Evidence level** | | | |
| --- | --- | --- | --- | --- | --- | --- |
|  |  |  | **Empagliflozin** | | **Linagliptin** | |
|  |  |  | **97.5%** | **95%** | **97.5%** | **95%** |
| 1 | 1 | ‒0.75 | 0.989 | 0.998 | 0.982 | 0.996 |
| 2 | 1 | ‒0.55 | 0.954 | 0.987 | 0.934 | 0.98 |
| 3 | 1 | ‒0.4 | 0.89 | 0.962 | 0.856 | 0.946 |
| 4 | 1 | 0 | 0.498 | 0.706 | 0.45 | 0.663 |
| 5 | 0.8 | ‒0.75 | 0.955 | 0.982 | 0.943 | 0.977 |
| 6 | 0.8 | ‒0.55 | 0.859 | 0.931 | 0.837 | 0.92 |
| 7 | 0.8 | ‒0.4 | 0.731 | 0.847 | 0.705 | 0.831 |
| 8 | 0.8 | 0 | 0.269 | 0.417 | 0.258 | 0.409 |
| 9 | 0.65 | ‒0.75 | 0.924 | 0.966 | 0.908 | 0.958 |
| 10 | 0.65 | ‒0.55 | 0.793 | 0.886 | 0.769 | 0.872 |
| 11 | 0.65 | ‒0.4 | 0.638 | 0.772 | 0.615 | 0.756 |
| 12 | 0.65 | 0 | 0.19 | 0.313 | 0.185 | 0.31 |
| 13 | 0.5 | ‒0.75 | 0.887 | 0.944 | 0.867 | 0.933 |
| 14 | 0.5 | ‒0.55 | 0.723 | 0.834 | 0.699 | 0.818 |
| 15 | 0.5 | ‒0.4 | 0.552 | 0.695 | 0.53 | 0.678 |
| 16 | 0.5 | 0 | 0.135 | 0.235 | 0.133 | 0.234 |
| 17 | 0 | ‒0.75 | 0.643 | 0.753 | 0.611 | 0.726 |
| 18 | 0 | ‒0.55 | 0.401 | 0.527 | 0.377 | 0.502 |
| 19 | 0 | ‒0.4 | 0.238 | 0.346 | 0.224 | 0.33 |
| 20 | 0 | 0 | 0.026 | 0.052 | 0.026 | 0.052 |

For prior means $\mu_{E}=\mu_{L}=-0.55$, an ESS of 100 per treatment group and informative prior weights $w_{E}=w_{L}=0.65$ the probability to meet the decision criterion with evidence level of 97.5% equals 79.3% (76.9%) for empagliflozin (linagliptin) in Scenario 10. Here, Scenario 10 corresponds to the placebo-corrected treatment effect assumed in the sample size calculation of DINAMO. This is a considerable improvement over the 40.1% (37.7%) probability in Scenario 18 where a weakly informative prior is used. If the placebo-corrected treatment effect in the empagliflozin group were ‒0.75 (as per Scenario 9), the probability to meet the decision criterion would increase to 92.4%. The use of the informative prior increases also the false decision probability. In Scenario 12, there is a 19.0% (18.5%) probability to falsely meet the decision criterion when there is no treatment benefit. This increase beyond the significance level chosen in the DINAMO protocol is considered acceptable. Overall, the operating characteristics for this choice of weights and ESS for the informative prior component provide a good balance between true and false decision probabilities.

**Table S6** Probability of meeting criterion for prior means for empagliflozin ($\mu_{E}$) and linagliptin ($\mu_{L}$) equal to 0 and informative part ESS of 100

| **Scenario**  **No.** | **Weight of**  **Informative part** | **True**  **treatment**  **effect in**  **children** | **Evidence level** | | | |
| --- | --- | --- | --- | --- | --- | --- |
|  |  |  | **Empagliflozin** | | **Linagliptin** | |
|  |  |  | **97.5%** | **95%** | **97.5%** | **95%** |
| 1 | 1 | ‒0.75 | 0.139 | 0.295 | 0.122 | 0.267 |
| 2 | 1 | ‒0.55 | 0.045 | 0.124 | 0.039 | 0.112 |
| 3 | 1 | ‒0.4 | 0.015 | 0.053 | 0.014 | 0.048 |
| 4 | 1 | 0 | 0 | 0.002 | 0 | 0.002 |
| 5 | 0.8 | ‒0.75 | 0.252 | 0.388 | 0.226 | 0.357 |
| 6 | 0.8 | ‒0.55 | 0.1 | 0.184 | 0.089 | 0.168 |
| 7 | 0.8 | ‒0.4 | 0.04 | 0.086 | 0.037 | 0.08 |
| 8 | 0.8 | 0 | 0.001 | 0.005 | 0.001 | 0.005 |
| 9 | 0.65 | ‒0.75 | 0.306 | 0.441 | 0.278 | 0.409 |
| 10 | 0.65 | ‒0.55 | 0.131 | 0.222 | 0.118 | 0.205 |
| 11 | 0.65 | ‒0.4 | 0.057 | 0.11 | 0.052 | 0.102 |
| 12 | 0.65 | 0 | 0.002 | 0.007 | 0.002 | 0.007 |
| 13 | 0.5 | ‒0.75 | 0.358 | 0.492 | 0.328 | 0.459 |
| 14 | 0.5 | ‒0.55 | 0.164 | 0.262 | 0.149 | 0.243 |
| 15 | 0.5 | ‒0.4 | 0.075 | 0.136 | 0.069 | 0.127 |
| 16 | 0.5 | 0 | 0.004 | 0.01 | 0.004 | 0.01 |
| 17 | 0 | ‒0.75 | 0.63 | 0.742 | 0.598 | 0.715 |
| 18 | 0 | ‒0.55 | 0.388 | 0.513 | 0.365 | 0.489 |
| 19 | 0 | ‒0.4 | 0.228 | 0.334 | 0.214 | 0.318 |
| 20 | 0 | 0 | 0.024 | 0.048 | 0.024 | 0.048 |

This shows the probability in the situation where the pharmacometrics model predicts no treatment benefit when there is a true benefit in children. Here, as expected, the probability of meeting the decision rules is low.

**Table S7** Structural, covariate and random effect parameter estimates from the final empagliflozin population pharmacokinetic model.

| **Parameter** | **Definition** | **Median (95% CDI)** | **ESS**^#^ | |
| --- | --- | --- | --- | --- |
|  |  |  | **Bulk** | **Tail** |
| **Fixed parameters** |  |  |  |  |
| CL/F [L/h] exp(*θ*_1_) | Apparent clearance after oral dosing | 8.45 (8.23‒8.67) | 4915 | 15228 |
| V_2_/F [L] exp(*θ*2) | Apparent central volume | 5.83 (5.12‒6.66) | 870 | 1829 |
| k_A_ [1/h] exp(*θ*3) | First order absorption rate constant | 0.237 (0.231‒0.244) | 710 | 1721 |
| Q/F [L/h] exp(*θ*4) | Apparent intercompartmental clearance | 5.59 (5.29‒5.90) | 653 | 1542 |
| V_3_/F [L/h] exp(*θ*5) | Apparent peripheral volume | 71.9 (68.1‒76.6) | 467 | 1183 |
| D1 (L) exp(*θ*6) | Zero order absorption duration | 0.297 (0.143‒0.399) | 1023 | 1671 |
| **Covariate effects on F** |  |  |  |  |
| WT_CL/F_ *θ*7 | Weight effect on CL/F | 0.75 (0.75‒0.75) | 40000 | 40000 |
| WT_V2/F_ *θ*8 | Weight effect on V_2_/F | 1.00 (1.00‒1.00) | 40000 | 40000 |
| WT_Q/F_ *θ*9 | Weight effect on Q/F | 0.75 (0.75‒0.75) | 40000 | 40000 |
| WT_V3/F_ *θ*10 | Weight effect on V_3_/F | 1.00 (1.00‒1.00) | 40000 | 40000 |
| **Covariate effects on CL/F** |  |  |  |  |
| EGFR_CL/F_ *θ*11 | eGFR effect on CL/F | 0.408 (0.362‒0.455) | 609 | 1307 |
| AGE_CL/F_ *θ*12 | Age effect on CL/F | -0.183 (-0.259‒-0.106) | 4774 | 14905 |
| BLACK_CL/F_ exp(*θ*13) | Race=Black effect on CL/F | 0.885 (0.817‒0.957) | 10384 | 19279 |
| ASIAN_CL/F_ exp(*θ*14) | Race=Asian effect on CL/F | 0.933 (0.902‒0.965) | 22840 | 33172 |
| FEMALE_CL/F_ exp(*θ*15) | Sex=Female effect on CL/F | 1.02 (0.985‒1.05) | 26946 | 34857 |

**Table S7** **cont.** Parameter estimates from the final empagliflozin population pharmacokinetic model.

| **Parameter** | **Definition** | **Median (95% CDI)** | **ESS**^#^ | |
| --- | --- | --- | --- | --- |
|  |  |  | **Bulk** | **Tail** |
| **Inter-individual variability** |  |  |  |  |
| IIV in CL/F (%) $\sqrt{e^{\omega_{11}-1}}\cdot100\%$ | Variance of CL/F | 55.2 (53.6‒56.9) [6.26%]* | 4358 | 14679 |
| IIV in V_3_/F (%)$\sqrt{e^{\omega_{11}-1}}\cdot100\%$ | Variance of V_3_/F | 40.5 (33.9‒48.4) [62.3%]* | 246 | 693 |
| V_3_/F-CL/F Ω_21_ | Covariance of V_3_/F - CL/F | 0.0837 (0.0575‒0.111) | 661 | 1715 |
| **Residual variability** |  |  |  |  |
| Proportional (%)$\sqrt{\varepsilon_{11}}\cdot100\%$ | Proportional RUV | 35.9 (35.4‒36.4) | 3095 | 5251 |
| Additive (%)$\sqrt{\varepsilon_{22}}$ | Additive RUV | 2.09 (1.98‒2.22) | 4407 | 6136 |

CDI, credible interval; ESS, effective sample size; IIV, interindividual variability (variance); RUV, residual, unexplained variability (variance); $\hat{R}$, Gelman-Rubin diagnostic.

Estimates (median [95% CDI]) for a typical subject (White, male, 70 kg, 55 years old, eGFR 90 mL/min/1.73m^2^) are shown.

The median and 95% credible intervals (CDIs) of parameter estimates were derived from the Bayesian posterior distribution of parameter estimates derived from the combined 40,000 sampling iterations across four chains (5000 burn-in iterations with 10,000 post burn-in samples per chain). The model used mu-referencing: estimates presented here were back-transformed from the log-domain for clarity.

^#^ESS was calculated using the method described by Vehtari and colleagues [41] .

*Shrinkage [%]

$\hat{R}$ (add formula) was 1.01 for V_3_/F, EGFR_CL/F_, IIV in V_3_/F, and V_3_/F-CL/F, and 1.00 for all other parameters.

**Table S8** Structural, covariate and random effect parameter estimates from the final empagliflozin exposure-response model.

| **Parameter** | **Definition** | **Estimate (95% CI)** |
| --- | --- | --- |
| **Fixed parameters** |  |  |
| K_OUT_ (day^−1^) exp(*θ*_1_) | HbA1c degradation rate constant | 0.0178 (0.0165‒0.0192) |
| BASE (%) exp(*θ*2) | Baseline HbA1c (%) | 8.17 (8.10‒8.23) |
| PBO (%) *θ*3 x 100% | Placebo effect on K_OUT_ | 5.16 (4.25‒6.07) |
| IMAX (%) exp(*θ*4) x 100% | Maximal inhibition of K_in_ | 10.9 (10.3‒11.6) |
| AUC_50_ (nmol**·**h/L) *θ*5 | AUC at 50% Imax | 703 (703‒703) (FIXED) |
| **Covariate parameters** |  |  |
| MET∼BASE exp(*θ*6) | Metformin effect on baseline | 1.00 (0.994, 1.01) |
| INS∼BASE *θ*_7_ | Insulin effect on baseline | 1.02 (1.01, 1.03) |
| SU∼BASE *θ*_8_ | Sulfonylurea effect on baseline | 1.01 (1.00, 1.02) |
| MET∼PBO *θ*9 | Metformin background effect on placebo effect | 1.97 (1.09, 2.85) |
| INS∼PBO *θ*10 x 100% | Insulin background effect on placebo effect | ‒1.22 (‒2.67, 0.233) |
| SU∼PBO *θ*11 x 100% | Sulfonylurea background effect on placebo effect | ‒2.49 (‒3.44, ‒1.54) |
| SEX∼BASE *θ*12 | Sex = Female effect on baseline HbA1c | 1.00 (0.995, 1.01) |
| AGE∼BASE *θ*13 | Body weight effect on baseline HbA1c | ‒0.0687 (‒0.0847, ‒0.0526) |
| WT∼BASE *θ*14 | Race = Black effect on baseline HbA1c | ‒0.0436 (‒0.0579, ‒0.0294) |
| BLACK∼BASE exp(*θ*15) | Race = Black effect on baseline HbA1c | 1.02 (1.01, 1.04) |
| ASIAN∼BASE exp(*θ*16) | Race = Asian effect on baseline HbA1c | 0.992 (0.985, 0.999) |
| EGFR∼BASE *θ*17 | eGFR effect on baseline HbA1c | 0.00306 (‒0.00745, 0.0136) |
| EGFR∼IMAX *θ*18 | eGFR effect on I_max_ | 1.04 (0.792, 1.30) |
| HBAB∼IMAX *θ*19 | Baseline HbA1c effect on I_max_ | 2.04 (1.46, 2.62) |

**Table S8 cont.** Structural, covariate and random effect parameter estimates from final empagliflozin exposure-response model

| **Parameter** | **Definition** | **Estimate (95% CI)** |
| --- | --- | --- |
| **Inter-individual variability** |  |  |
| IIV-K_OUT_ $\sqrt{e^{\omega_{11}-1}}\cdot100\%$ | Variance of k_out_ | 171 (153, 191) [47.4]* |
| COV-K_OUT_∼BASE | Covariance of kout∼baseline HbA1c | 0.00993 (0.00357, 0.0163) |
| IIV-BASE $\sqrt{e^{\omega_{22}-1}}\cdot100\%$ | Variance of baseline HbA1c | 10.7 (10.4, 10.9) [4.63]* |
| COV-PBO∼K_OUT_ | Covariance of placebo effect∼k_out_ | 0.0183 (0.00935, 0.0273) |
| COV-PBO∼BASE | Covariance of placebo effect∼baseline HbA1c | 0.00541 (0.00444, 0.00637) |
| IIV-PBO $\sqrt{e^{\omega_{33}}}\cdot100\%$ | Standard deviation of placebo effect | 13.1 (12.5, 13.7) [13.9]* |
| IIV-IMAX $\sqrt{e^{\omega_{44}-1}}\cdot100\%$ | Variance of maximal inhibition | 10.0 (10.0, 10.0) [89.6]* |
| **Residual variability** |  |  |
| Log-Additive (%)$\sqrt{e^{\varepsilon_{11}-1}}\cdot100\%$ | Additive RUV on log scale | 5.03 (4.90, 5.16) |

CI, confidence interval; IIV: inter-individual variability (variance); IOV: inter-occasion variability (variance); RUV: residual, unexplained variability (variance)

Estimates (median [95% CDI]) for a typical subject (white, male, 70 kg, 55 years old, eGFR 90 mL/min/1.73m^2^, baseline HbA1c 7.9%, and not taking concomitant metformin, sulfonylureas, or insulin) are shown.

95% CIs of parameter estimates were calculated from the standard error estimates.

*Shrinkage [%].

**Table S9** Parameter estimates from the final linagliptin population pharmacokinetic model.

| **Parameter** | **Definition** | **Estimate** | **Median (95% CI)** | **Shrinkage (%)** |
| --- | --- | --- | --- | --- |
| **Fixed effect parameters** | |  |  |  |
| CL/F [L/h] *θ*_1_ | Apparent clearance | 153 | 151 (133‒181) |  |
| V_2_/F [L] *θ*2 | Apparent central volume of distribution | 788 | 784 (657‒893) |  |
| Q_3_/F [L/h] *θ*3 | Apparent intercompartmental clearance | 222 | 222 (177‒284) |  |
| V_3_/F [L] *θ*4 | Apparent peripheral volume of distribution | 1340 | 1350 (1170‒1530) |  |
| k_A_ [1/h] *θ*6 | Absorption rate constant | 0.844 | 0.840 (0.682‒0.969) |  |
| KSS (nmol/L) *θ*7 | DPP-4 binding affinity | 0.0624 | 0.0620 (0.0515‒0.0717) |  |
| RMAX (nmol/L) *θ*8 | Total DPP-4 concentration | 4.09 | 4.08 (3.74‒4.40) |  |
| **Covariate effects** |  |  |  |  |
| CL/F~SEX *θ*7 | Proportional shift in CL/F for female relative to male | 0.844 | 0.841 (0.759‒0.965) |  |
| **Inter-individual variability** | |  |  |  |
| Ω_CL/F_  Ω_11_ | Variance of CL/F | 0.270 | 0.269 (0.139‒0.427) | 12 |
| Ω_KA_ Ω_22_ | Variance of K_A_ | 0.750 | 0.752 (0.549‒0.973) | 25 |
| Ω_IOV-F_ Ω_33_ | Variance of F1 | 0.351 | 0.335 (0.241‒0.523) | 26.4 |
| **Residual variability** |  |  |  |  |
| Ω_1218.2,3_ Σ_11_ | RUV of trials 1218.2 [7] and 1218.3 [8] | 0.0218 | 0.0217 (0.0192‒0.0245) | 5.8 |
| Ω_others_ Σ_22_ | RUV of trials 1218.5 [9], 1218.6 [10] and 1218.56 [11] | 0.129 | 0.129 (0.0978‒0.163) | 16.4 |

CDI, credible interval: ESS, effective sample size: IIV, interindividual variability.

The median and 95% CDIs of parameter estimates were derived from a non-parametric bootstrap. The model used mu-referencing: estimates presented here were back-transformed from the log-domain for clarity.

**Table S10** Parameter estimates from the final linagliptin exposure-response model.

| **Parameter** | **Definition** | **Estimate** | **Median (95% CI)** |
| --- | --- | --- | --- |
| **Fixed parameters** |  |  |  |
| BASE exp(*θ*_1_) | HbA1c at start of therapy (%) | 7.95 | 7.95 (7.91‒8.00) |
| KIN exp(*θ*_2_) | HbA1c zero order input (% per hour) | 0.0105 | 0.0105 (0.00840‒0.0130) |
| WOEFF exp(*θ*_3_) | Washout effect (% per hour) | 0.000427 | 0.000425 (0.000310‒0.000553) |
| IMAX exp(*θ*_6_)/1+ exp(*θ*_6_) | Maximum inhibitory effect of linagliptin | 0.141 | 0.141 (0.111‒0.200) |
| AUC_50_ (nmol**·**h/L) *θ*_7_ | Linagliptin AUC_ss_ for half-maximal inhibition | 136 | 135 (66.8‒274) |
| **Covariate effects** |  |  |  |
| METKIN exp(*θ*_4_) | Metformin effect on baseline | 0.965 | 0.970 (0.804‒1.18) |
| INSKIN exp(*θ*_5_) | Insulin effect on baseline | 1.22 | 1.21 (0.973‒1.56) |
| METBASE exp(*θ*_8_) | Sulfonylurea effect on baseline | 0.985 | 0.985 (0.978‒0.993) |
| INBASE exp(*θ*_9_) | Metformin background effect on placebo effect | 1.05 | 1.05 (1.04‒1.06) |
| **Variance estimates** |  |  |  |
| Ω_BASE_ Ω_11_ | IIV - BASE | 0.0112 | 0.0111 (0.0106‒0.0117) |
| Σ_11_ Σ11 | RUV - exponential | 0.00338 | 0.00337 (0.00318‒0.00358) |

IIV, inter-individual variability (variance): RUV, residual, unexplained variability (variance)

The median and 95% CIs of parameter estimates were derived from a non-parametric bootstrap.

eta-shrinkage: 3.1%, epsilon-shrinkage: 10.1%.

**Figure S1** Simulated distribution of the mean placebo-corrected treatment effect for empagliflozin (**A**) and linagliptin (**B**) in a population of 5,000 pediatric patients.


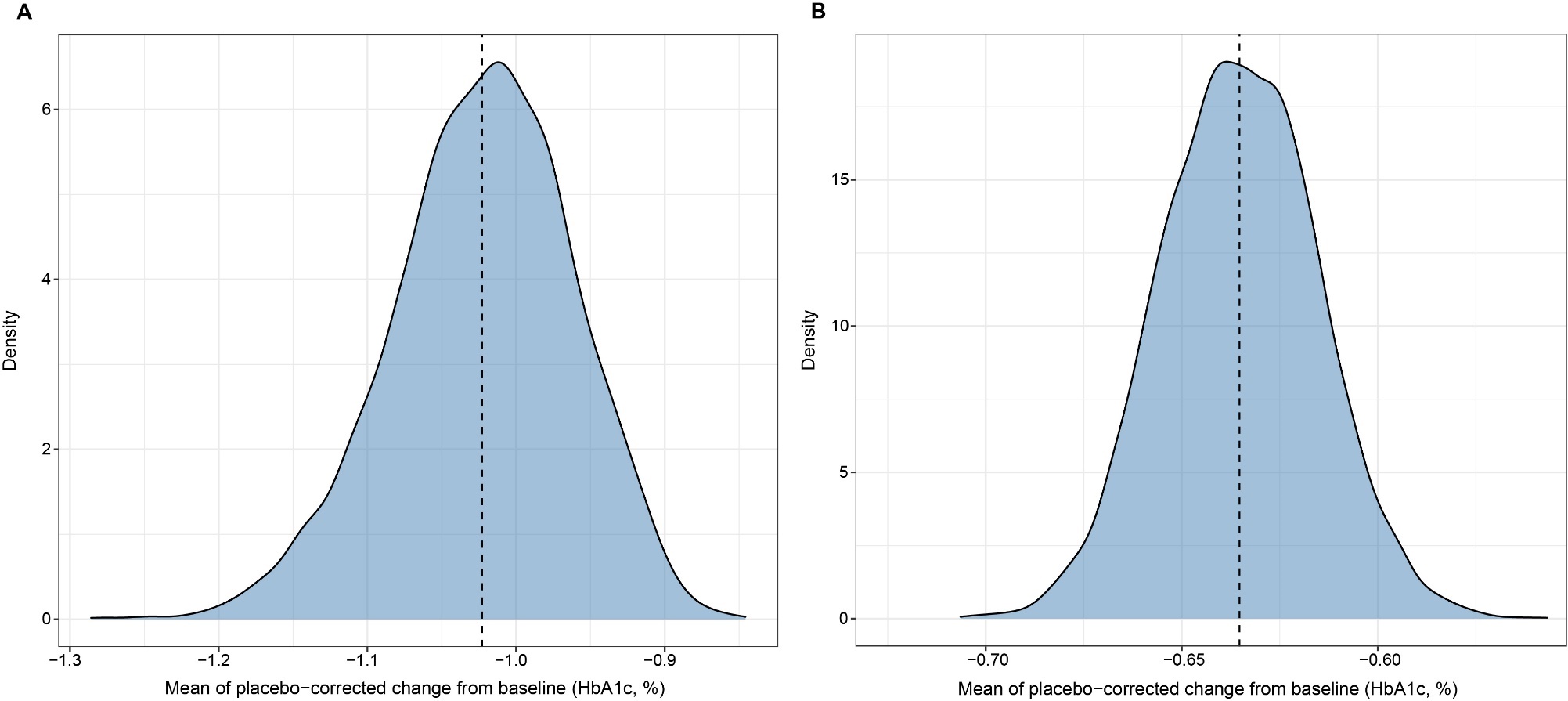


The simulations were conducted in an iterative manner for a total of 5,000 iterations. In each iteration, 5,000 patients per treatment group (linagliptin and empagliflozin). were resampled without stratification using the blinded demographic and background medication data from DINAMO (158 randomized participants). Longitudinal HbA1c was simulated for each simulated patient, including patient-level random effects. Each trial was summarized with the mean placebo-corrected treatment effect shown by the vertical dotted lines.

**Figure S2** Power and type I error for empagliflozin (**A**) and linagliptin (**B**) for the final priors used in the analysis as a function of the unit-information SD for the placebo-corrected treatment effects in the DINAMO trial.

| **A** | **B** |
| --- | --- |
| 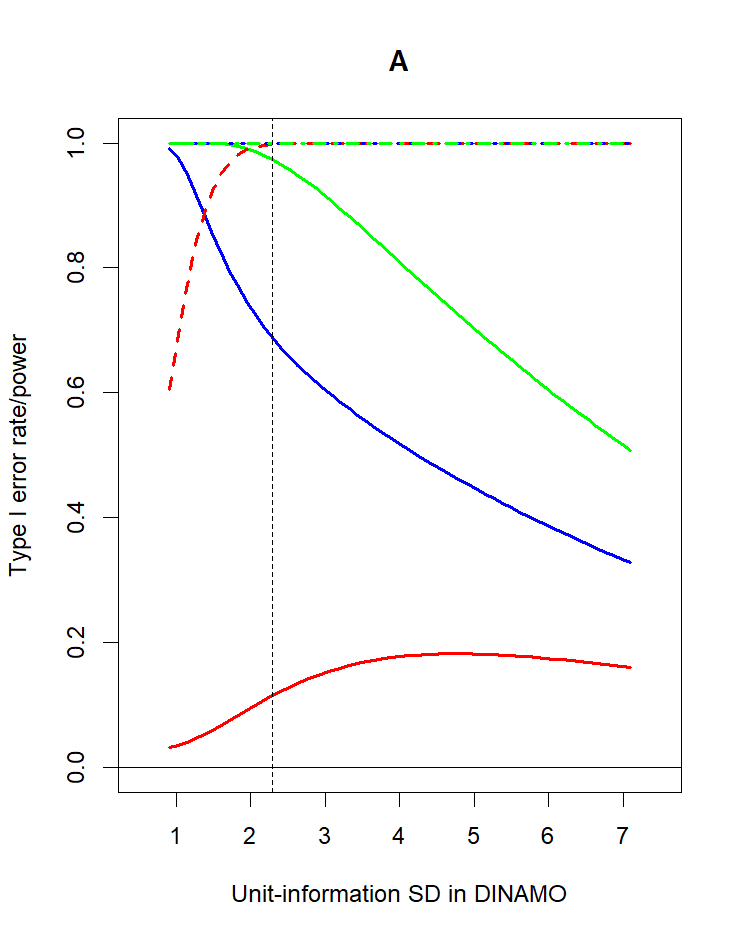 | 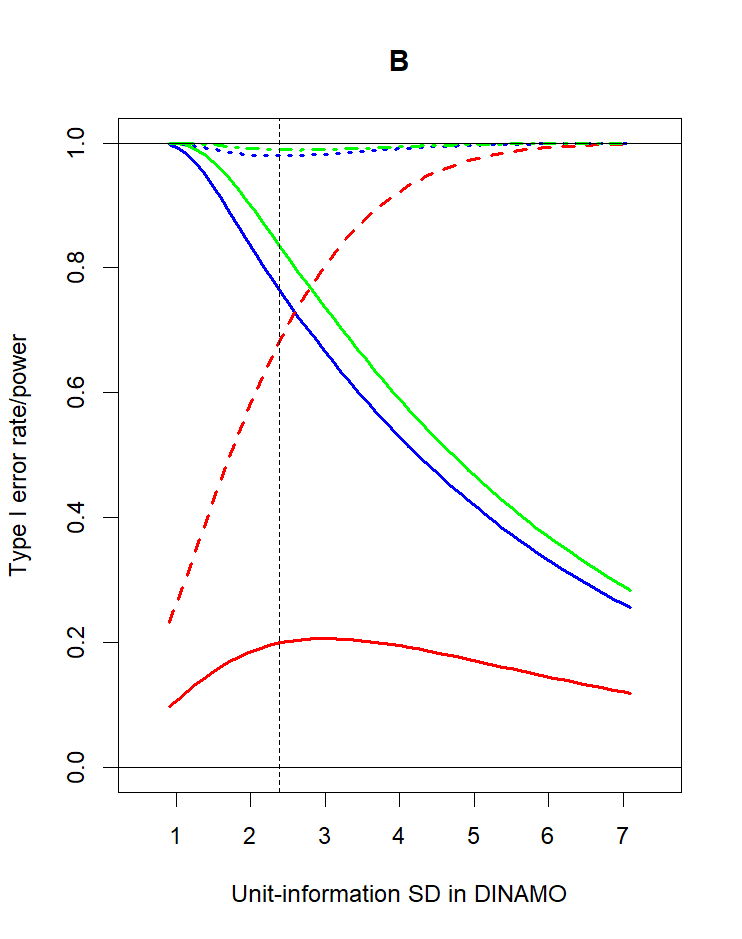 |

Type I error (red) and power for assumed true placebo-corrected treatment effects of ‒0.55 (blue) and treatment effects corresponding to the prior mean (green) as a function of the unit-information SD for the placebo-corrected treatment effects in the DINAMO trial. Here, the decision rules with evidence level 97.5% are used. The vertical black dashed line corresponds to the SD in the final analysis prior. Solid lines correspond to the robust prior used in the DINAMO analysis. Broken lines correspond to a prior with informative prior weight of 1 (i.e. without robustification). The robust prior limits the type I error inflation at the price of a reduction in power. The power remains high as long as there is no substantial prior-data conflict.

**Figure S3** Impact of potential bias introduced through informative priors on the power of the analysis of the placebo-corrected treatment effects of empagliflozin (**A**) and linagliptin (**B**).

| **A** | **B** |
| --- | --- |
| 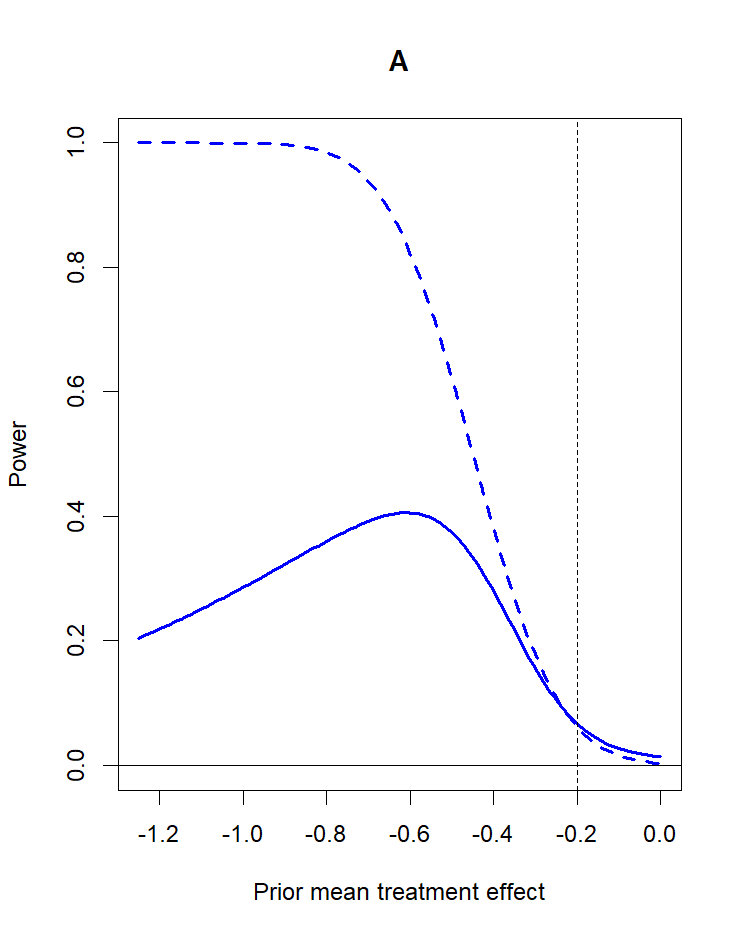 | 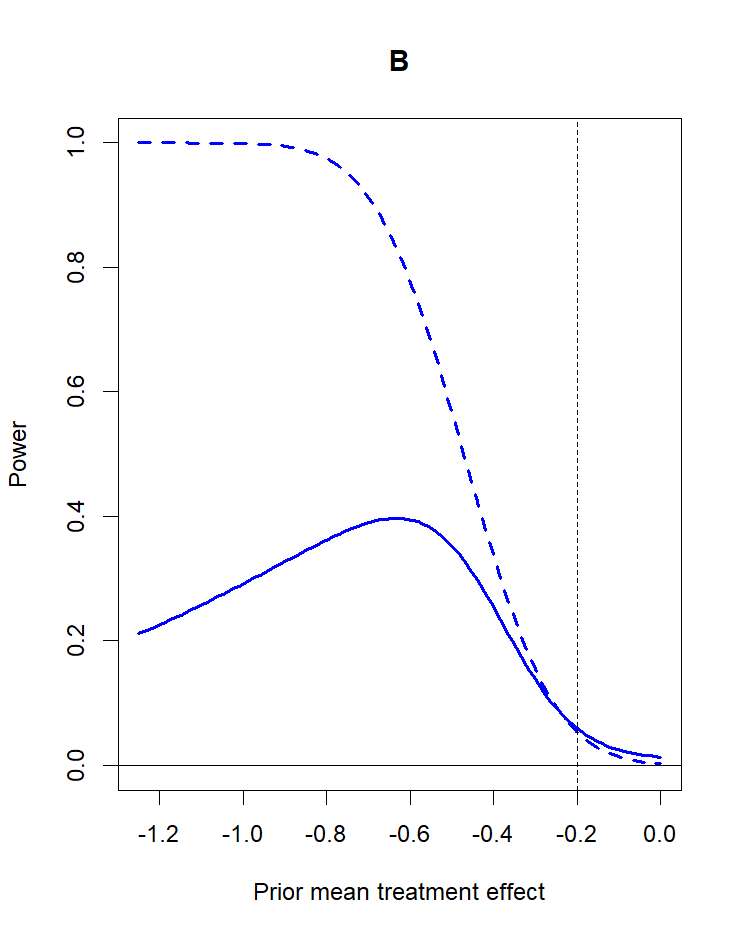 |

Power for declaring placebo-corrected treatment effects as a function of the potential bias in the pharmacometric model simulations. Bias is defined as the prior mean minus the true placebo-corrected treatment effect in DINAMO. Decision rules with evidence level 97.5% are used. To illustrate a potential increase in power in a situation of marginal treatment benefit, a true placebo corrected mean of ‒0.20 is assumed. The solid blue line corresponds to robust priors with weight of 0.65 for the informative part of the prior. The dashed blue line corresponds to a non-robust prior with an informative weight of 1. If the pharmacometric model simulations overestimate the treatment effect, the power would increase. Here, this potential increase is limited to 40.6% (39.7%) for the robust prior while it is unbounded for non-robust borrowing.

# **SUPPLEMENTAL REFERENCES**

1. Beal SL, Sheiner LB, Boeckmann AJ. NONMEM Users Guide: Part I-VII: Icon Development Solutions, Ellicott City, Maryland, USA.

2. R Core Team. R: A language and environment for statistical computing. R Foundation for Statistical Computing, Vienna, Austria. <http://www.r-project.org/index.html>. Accessed May 14, 2024.

3. Weber S, Li Y, Seaman JW, et al. Applying meta-analytic-predictive priors with the R Bayesian evidence synthesis tools. J Statist Software. 2021;100(19):1-32.

4. Riggs MM, Staab A, Seman L, et al. Population pharmacokinetics of empagliflozin, a sodium glucose cotransporter 2 inhibitor, in patients with type 2 diabetes. J Clin Pharmacol. 2013;53(10):1028-38.

5. Hüttner S, Graefe-Mody EU, Withopf B, et al. Safety, tolerability, pharmacokinetics, and pharmacodynamics of single oral doses of BI 1356, an inhibitor of dipeptidyl peptidase 4, in healthy male volunteers. J Clin Pharmacol. 2008;48(10):1171-8.

6. Retlich S, Duval V, Graefe-Mody U, et al. Impact of target-mediated drug disposition on Linagliptin pharmacokinetics and DPP-4 inhibition in type 2 diabetic patients. J Clin Pharmacol. 2010;50(8):873-85.

7. Boehringer Ingelheim. Safety, tolerability, pharmacokinetics and pharmacodynamics of BI 1356 BS in patients with type 2 diabetes. Trial no. 1218.2. <https://www.mystudywindow.com/trial/completed/192913/1218-0002>. Accessed May 14, 2024.

8. Boehringer Ingelheim. Safety, tolerability, pharmacokinetics and pharmacodynamics of BI 1356 BS as tablet in patients with type 2 diabetes. Trial no. 1218.3. <https://www.mystudywindow.com/trial/completed/192915/1218-0003>. Accessed May 14, 2024.

9. Boehringer Ingelheim. Efficacy and safety of 3 doses of BI1356 (linagliptin) in type 2 diabetes patients. Trial no. 1218.5. <https://www.mystudywindow.com/trial/completed/192565/1218-0005>. Accessed May 14, 2024.

10. Boehringer Ingelheim. Efficacy and safety of BI 1356 BS (linagliptin) in combination with metformin in patients with type 2 diabetes. Trial no. 1218.6. <https://www.mystudywindow.com/trial/completed/192561/1218-0006>. Accessed May 14, 2024.

11. Boehringer Ingelheim. Finding a safe and effective dose of linagliptin in pediatric patients with type 2 diabetes. Trial no. 1218.56. <https://www.mystudywindow.com/trial/completed/192739/1218-0056>. Accessed May 14, 2024.

12. Retlich S, Duval V, Graefe-Mody U, et al. Population pharmacokinetics and pharmacodynamics of linagliptin in patients with type 2 diabetes mellitus. Clin Pharmacokinet. 2015;54(7):737-50.

13. Tamborlane WV, Laffel LM, Weill J, et al. Randomized, double-blind, placebo-controlled dose-finding study of the dipeptidyl peptidase-4 inhibitor linagliptin in pediatric patients with type 2 diabetes. Pediatr Diabetes. 2018;19(4):640-8.

14. Del Prato S, Barnett AH, Huisman H, et al. Effect of linagliptin monotherapy on glycaemic control and markers of β-cell function in patients with inadequately controlled type 2 diabetes: a randomized controlled trial. Diabetes Obes Metab. 2011;13(3):258-67.

15. Gallwitz B, Rosenstock J, Rauch T, et al. 2-year efficacy and safety of linagliptin compared with glimepiride in patients with type 2 diabetes inadequately controlled on metformin: a randomised, double-blind, non-inferiority trial. Lancet. 2012;380(9840):475-83.

16. Yki-Järvinen H, Rosenstock J, Durán-Garcia S, et al. Effects of adding linagliptin to basal insulin regimen for inadequately controlled type 2 diabetes: a ≥52-week randomized, double-blind study. Diabetes Care. 2013;36(12):3875-81.

17. Yao Z, Krzyzanski W, Jusko WJ. Assessment of basic indirect pharmacodynamic response models with physiological limits. J Pharmacokinet Pharmacodyn. 2006;33(2):167-93.

18. Riggs MM, Seman LJ, Staab A, et al. Exposure-response modelling for empagliflozin, a sodium glucose cotransporter 2 (SGLT2) inhibitor, in patients with type 2 diabetes. Br J Clin Pharmacol. 2014;78(6):1407-18.

19. Parkinson J, Tang W, Åstrand M, et al. Model-based characterization of the relationship between dapagliflozin systemic exposure and HbA1c response in patients with type 1 diabetes mellitus. Diabetes Obes Metab. 2019;21(6):1381-7.

20. Yano Y, Beal SL, Sheiner LB. Evaluating pharmacokinetic/pharmacodynamic models using the posterior predictive check. J Pharmacokinet Pharmacodyn. 2001;28(2):171-92.

21. Neuenschwander B, Schmidli H. Use of historical data. In: Lesaffre E, Baio G, Boulanger B, editors. *Bayesian Methods in Pharmaceutical Research*. First ed. New York: Chapman & Hall / CRC; 2020. p. 111-37.

22. Häring HU, Merker L, Seewaldt-Becker E, et al. Empagliflozin as add-on to metformin plus sulfonylurea in patients with type 2 diabetes: a 24-week, randomized, double-blind, placebo-controlled trial. Diabetes Care. 2013;36(11):3396-404.

23. Rosenstock J, Jelaska A, Zeller C, et al. Impact of empagliflozin added on to basal insulin in type 2 diabetes inadequately controlled on basal insulin: a 78-week randomized, double-blind, placebo-controlled trial. Diabetes Obes Metab. 2015;17(10):936-48.

24. Rosenstock J, Jelaska A, Frappin G, et al. Improved glucose control with weight loss, lower insulin doses, and no increased hypoglycemia with empagliflozin added to titrated multiple daily injections of insulin in obese inadequately controlled type 2 diabetes. Diabetes Care. 2014;37(7):1815-23.

25. Taskinen MR, Rosenstock J, Tamminen I, et al. Safety and efficacy of linagliptin as add-on therapy to metformin in patients with type 2 diabetes: a randomized, double-blind, placebo-controlled study. Diabetes Obes Metab. 2011;13(1):65-74.

26. Schmidli H, Gsteiger S, Roychoudhury S, et al. Robust meta-analytic-predictive priors in clinical trials with historical control information. Biometrics. 2014;70(4):1023-32.

27. Neuenschwander B, Weber S, Schmidli H, et al. Predictively consistent prior effective sample sizes. Biometrics. 2020;76(2):578-87.

28. Travis J, Rothmann M, Thomson A. Perspectives on informative Bayesian methods in pediatrics. J Biopharm Stat. 2023;33(6):830-43.

29. Heise T, Seman L, Macha S, et al. Safety, tolerability, pharmacokinetics, and pharmacodynamics of multiple rising doses of empagliflozin in patients with type 2 diabetes mellitus. Diabetes Ther. 2013;4(2):331-45.

30. Heise T, Seewaldt-Becker E, Macha S, et al. Safety, tolerability, pharmacokinetics and pharmacodynamics following 4 weeks' treatment with empagliflozin once daily in patients with type 2 diabetes. Diabetes Obes Metab. 2013;15(7):613-21.

31. Ferrannini E, Seman L, Seewaldt-Becker E, et al. A Phase IIb, randomized, placebo-controlled study of the SGLT2 inhibitor empagliflozin in patients with type 2 diabetes. Diabetes Obes Metab. 2013;15(8):721-8.

32. Rosenstock J, Seman LJ, Jelaska A, et al. Efficacy and safety of empagliflozin, a sodium glucose cotransporter 2 (SGLT2) inhibitor, as add-on to metformin in type 2 diabetes with mild hyperglycaemia. Diabetes Obes Metab. 2013;15(12):1154-60.

33. Kanada S, Koiwai K, Taniguchi A, et al. Pharmacokinetics, pharmacodynamics, safety and tolerability of 4 weeks' treatment with empagliflozin in Japanese patients with type 2 diabetes mellitus. J Diabetes Investig. 2013;4(6):613-7.

34. Kovacs CS, Seshiah V, Swallow R, et al. Empagliflozin improves glycaemic and weight control as add-on therapy to pioglitazone or pioglitazone plus metformin in patients with type 2 diabetes: a 24-week, randomized, placebo-controlled trial. Diabetes Obes Metab. 2014;16(2):147-58.

35. Roden M, Weng J, Eilbracht J, et al. Empagliflozin monotherapy with sitagliptin as an active comparator in patients with type 2 diabetes: a randomised, double-blind, placebo-controlled, phase 3 trial. Lancet Diabetes Endocrinol. 2013;1(3):208-19.

36. Ridderstråle M, Andersen KR, Zeller C, et al. Comparison of empagliflozin and glimepiride as add-on to metformin in patients with type 2 diabetes: a 104-week randomised, active-controlled, double-blind, phase 3 trial. Lancet Diabetes Endocrinol. 2014;2(9):691-700.

37. Barnett AH, Mithal A, Manassie J, et al. Efficacy and safety of empagliflozin added to existing antidiabetes treatment in patients with type 2 diabetes and chronic kidney disease: a randomised, double-blind, placebo-controlled trial. Lancet Diabetes Endocrinol. 2014;2(5):369-84.

38. Laffel LMB, Tamborlane WV, Yver A, et al. Pharmacokinetic and pharmacodynamic profile of the sodium-glucose co-transporter-2 inhibitor empagliflozin in young people with Type 2 diabetes: a randomized trial. Diabet Med. 2018;35(8):1096-104.

39. Hadjadj S, Rosenstock J, Meinicke T, et al. Initial combination of empagliflozin and metformin in patients with type 2 diabetes. Diabetes Care. 2016;39(10):1718-28.

40. Boehringer Ingelheim. A 16-week study on efficacy and safety of two doses of empagliflozin (BI 10773) (once daily versus twice daily) in patients with type 2 diabetes mellitus and preexisting metformin therapy. Trial no. 1276.10. <https://www.mystudywindow.com/trial/completed/192775/1276-0010>. Accessed May 14, 2024.

41. Vehtari A, Gelman A, Simpson D, et al. Rank-normalization, folding, and localization: An improved $\hat{R}$ for assessing convergence of MCMC (with discussion). Bayesian Analysis. 2021;16(2):667-718.
